# Integration of anatomy ontology data with protein–protein interaction networks improves the candidate gene prediction accuracy for anatomical entities

**DOI:** 10.1186/s12859-020-03773-2

**Published:** 2020-10-07

**Authors:** Pasan C. Fernando, Paula M. Mabee, Erliang Zeng

**Affiliations:** 1 Department of Biology, University of South Dakota, Vermillion, SD USA; 2 Division of Biostatistics and Computational Biology, College of Dentistry, University of Iowa, Iowa City, IA USA; 3 Department of Preventive and Community Dentistry, College of Dentistry, University of Iowa, Iowa City, IA USA; 4 Department of Biostatistics, College of Public Health, University of Iowa, Iowa City, IA USA; 5 Department of Biomedical Engineering, College of Engineering, University of Iowa, Iowa City, IA USA; 6 Present Address: National Ecological Observatory Network, Battelle Memorial Institute, 1685 38th St., Suite 100, Boulder, CO 80301 USA

**Keywords:** Anatomy ontology, Uberon, Phenotype, Protein–protein interaction networks, Big data, Data integration, Candidate gene prediction, Data quality, Semantic similarity

## Abstract

**Background:**

Identification of genes responsible for anatomical entities is a major requirement in many fields including developmental biology, medicine, and agriculture. Current wet lab techniques used for this purpose, such as gene knockout, are high in resource and time consumption. Protein–protein interaction (PPI) networks are frequently used to predict disease genes for humans and gene candidates for molecular functions, but they are rarely used to predict genes for anatomical entities. Moreover, PPI networks suffer from network quality issues, which can be a limitation for their usage in predicting candidate genes. Therefore, we developed an integrative framework to improve the candidate gene prediction accuracy for anatomical entities by combining existing experimental knowledge about gene-anatomical entity relationships with PPI networks using anatomy ontology annotations. We hypothesized that this integration improves the quality of the PPI networks by reducing the number of false positive and false negative interactions and is better optimized to predict candidate genes for anatomical entities. We used existing Uberon anatomical entity annotations for zebrafish and mouse genes to construct gene networks by calculating semantic similarity between the genes. These anatomy-based gene networks were semantic networks, as they were constructed based on the anatomy ontology annotations that were obtained from the experimental data in the literature. We integrated these anatomy-based gene networks with mouse and zebrafish PPI networks retrieved from the STRING database and compared the performance of their network-based candidate gene predictions.

**Results:**

According to evaluations of candidate gene prediction performance tested under four different semantic similarity calculation methods (Lin, Resnik, Schlicker, and Wang), the integrated networks, which were semantically improved PPI networks, showed better performances by having higher area under the curve values for receiver operating characteristic and precision-recall curves than PPI networks for both zebrafish and mouse.

**Conclusion:**

Integration of existing experimental knowledge about gene-anatomical entity relationships with PPI networks via anatomy ontology improved the candidate gene prediction accuracy and optimized them for predicting candidate genes for anatomical entities.

## Background

Unraveling the molecular function and associated phenotypes of proteins is a cornerstone in molecular biology. In particular, understanding the genes associated with the formation of anatomical structures, also termed ‘anatomical entities’**,** is essential in developmental biology [1–4]. The majority of genes associated with anatomical entities are obtained using wet lab methods, such as gene knockout [5, 6], gene knockdown [7], and overexpression [8, 9]. These methods, however, are time-consuming and require significant resources; thus only a few genes may be associated with the development of a particular anatomical entity, though there are likely more genes involved.

Alternatively, computational prediction methods for discovering gene-anatomical entity associations are employed because of their higher speed and low resource consumption [10, 11]. Sequence similarity-based function prediction is such an example, which is widely used to predict the molecular functions of proteins [12, 13]. However, using this method to predict the anatomical associations of genes is questionable, because anatomical entities develop from a combination of several biological pathways that include proteins with diverse molecular functions and sequences [14]. On the other hand, protein–protein interaction (PPI) networks can be used to predict candidate genes for anatomical entities, based on the assumption that proteins that regulate the same anatomical entity or function are more likely to physically interact with each other [15–17]. PPI networks represent such interactions as graphs where proteins are represented by nodes and their interactions are represented by edges. PPI networks have been widely used in predicting candidate genes for human disease phenotypes [18–20], but rarely used for predicting candidate genes associated with anatomical entities [17]. The main challenge with PPI network-based candidate gene prediction is to improve the accuracy of the predictions [15, 21–24], which are low because of the poor quality of large-scale PPI network data sets [16, 24–26]. PPI networks are generated by experimental methods, such as yeast two-hybrid assay and high-throughput mass-spectrometric protein complex identification, which can generate false positive interactions [22]. Furthermore, PPI networks for model organisms are still incomplete and the quality of data varies depending on the model [21, 27]. For instance, well studied organisms such as human and mouse contain more complete PPI network data sets compared to *Xenopus* or zebrafish [16, 23].

The STRING database is the most widely used PPI database, and it currently contains PPI networks for 5090 (05/15/2020) organisms [21, 28]. To improve the quality of PPI network data, the STRING database also computationally predicts the strength of an interaction between two proteins based on properties such as co-expression in addition to experimental evidence. This results in additional quality-controlled PPI network datasets. However, the STRING database does not incorporate experimental evidence regarding proteins that are regulating similar anatomical entities, which motivated us to integrate such experimental evidence based on the assumption that two proteins regulating similar anatomical entities are more likely to interact with each other.

The information regarding gene-anatomical entity associations that are discovered via wet lab techniques, such as gene knockout, is recorded in literature, annotated to model organism databases using anatomy ontologies and is available through databases and integrated repositories such as the Monarch Initiative [29, 30]. The anatomical entity associations of genes in the Monarch Initiative repository are annotated using Uberon anatomy ontology entities [31–33]. Such ontology annotations enable the calculation of the functional similarity between any two genes using computational methods [34–37]. By calculating the pairwise similarity for all the gene pairs using the anatomical annotations for each gene, a semantic gene network can be constructed. Such a network is referred to as an ‘anatomy-based gene network’.

The concept of constructing gene networks using ontology information such as the Gene Ontology (GO) has been previously presented [20, 38–40]. For example, Jiang, et al. [39] constructed a gene network using the Gene Ontology-Biological Process (GO-BP) entities to infer disease genes in humans. This gene network, however, was not integrated with an existing PPI network; instead, it was used directly for disease gene prediction, and the results were compared with a human PPI network. They discovered that the semantic gene network outperformed the PPI network for predicting disease genes [39]. Zeng et al. [38] performed gene function prediction using both PPI data and GO annotations, where the semantic similarity between GO terms was used to derive semantic similarity between genes, which was then used to evaluate the quality of PPI data [38]. This was based on the assumption that semantic similarity between genes serves as a metric of support for PPI data [41], which further could be used to predict linkages between genes to generate a functional gene network and reduce the false positive and false negative interactions in the PPI networks to improve their network quality [42]. On the other hand, PPI networks were rarely used to predict candidate genes for anatomical entities in the literature. One rare example was the work by Wang, et al. [17], in which they assessed the PPI network-based candidate gene prediction performance for phenotypes, some of which were associated with anatomical entities for six organisms, including mouse and zebrafish. They successfully demonstrated the usage of PPI networks for predicting anatomical phenotypes of proteins by predicting Zebrafish Anatomy Ontology [43] entities for zebrafish proteins and Mammalian Phenotype Ontology [44] entities for mouse proteins. However, more research was essential to accurately predict candidate genes for anatomical entities.

Motivated by the lack of previous attempts to predict candidate genes for anatomical entities and with the goal to improve the accuracy of such predictions, here, we extended the applicability of PPI networks for predicting gene-anatomical entity associations by integrating semantic networks constructed using existing associations with PPI networks. To our knowledge, this was the first work that used semantic networks constructed using an anatomy ontology. We hypothesized that this integration would improve the candidate gene prediction accuracy for anatomical entities, such as limbs and fins, by improving the quality of the original PPI networks. To test this hypothesis, we constructed semantic similarity gene networks based on anatomy ontology annotations (anatomy-based gene networks) using multiple semantic similarity methods (Additional file 1: Fig. S1) and integrated them with the PPI networks retrieved from the STRING database. Then, we evaluated the candidate gene prediction performances of the networks to confirm whether the integration improved the candidate gene prediction accuracy for anatomical entities.

## Results

### Data sources

The raw zebrafish STRING PPI network (01/02/2018) contained 23,018 genes and 12,558,675 interactions and the raw mouse STRING PPI network (01/02/2018) contained 21,052 genes and 6,262,253 interactions. After applying the 0.7 gene similarity score cutoff to keep only the high-quality interactions, the filtered zebrafish PPI network contained 14,677 genes and 501,704 interactions, and the filtered mouse PPI network contained 13,866 genes and 414,667 interactions.

The original zebrafish anatomical profiles retrieved from the Monarch Initiative repository (01/08/2018) contained 5405 genes annotated to 960 anatomical entities, and the mouse profiles contained 14,652 genes annotated to 1537 anatomical entities (Table 1). Not all of these genes were found in the STRING PPI networks, owing to differences in the data sources (2878 mismatches for zebrafish and 6486 mismatches for mouse; Table 1). After implementing the gene reconciliation algorithm that contained three rounds (direct name matching, Ensembl ID matching, and gene synonym matching), the number of original matches was increased from 2527 (direct name matching) to 3048 for the zebrafish and from 8166 (direct name matching) to 8607 for the mouse (Table 1). The detailed reconciliation statistics are shown in Table 1.

**Table 1:** The statistics for the reconciliation of gene names between the anatomical profiles from the Monarch, and the PPI networks from STRING for zebrafish and mouse

	Zebrafish	Mouse
Number of genes in the original anatomical profiles	5405	14,652
Number of anatomical entities in the original anatomical profiles	960	1537
Number of genes directly matched to the PPI network using the gene name (round 1)	2527	8166
Number of genes matched using the Ensembl IDs (round 2)	402	378
Number of genes matched using synonyms in the STRING database (round 3)	119	63
Number of final gene matches to the PPI network (in all 3 rounds)/Number of genes in the reconciled anatomical profiles	3048	8607
Number of anatomical entities in the reconciled anatomical profiles	943	1524
Number of final gene mismatches	2357	6045
Number of genes kept in the anatomical profiles after filtering out the entities with fewer than 10 gene annotations	3037	8606
Number of anatomical entities kept in the anatomical profiles after filtering out the entities with fewer than 10 gene annotations	294	850

The extra 521 genes for the zebrafish and 441 genes for the mouse that were matched during reconciliation round 2 (using Ensembl IDs) and round 3 (using gene synonyms) contained outdated gene names in the PPI networks. Therefore, they were updated to the correct names that were used in the anatomical profiles. The final number of gene mismatches for zebrafish and mouse were 2357 and 6045, respectively. The majority of these mismatched genes had anatomical term annotations in the Monarch Initiative repository, but their proteins were not characterized and investigated in the literature. Therefore, there were no protein interaction records in the STRING database. After the reconciliation, the original anatomical profiles were filtered to contain only the genes that were matched with the PPI networks and to contain only the anatomical entities that had at least 10 gene annotations (Table 1). We selected 10 gene annotations because it had been proven that evaluation using smaller datasets led to higher error rates [59, 60]. To further investigate this, we compared the AUC distributions of ROC curves for anatomical entities with less than 10 annotations versus anatomical entities with 10–100 and above 100 annotations generated using the zebrafish PPI network (Additional file 1: Fig. S2). According to the results in the violin plots, the anatomical entities with less than 10 annotations showed a higher variation in AUC values ranging from 0–1 with the peak of the distribution at 0.3. In comparison, the AUC distributions for anatomical entities with more than 10 annotations were more stable with an AUC peak value around 0.7. This showed the instability of the prediction performances when using anatomical entities with less than 10 annotations, justifying the selection of anatomical entities with at least 10 annotations for a reliable evaluation.

These reconciled and filtered anatomical profiles were used during the evaluation because it was important to evaluate the networks using the genes that were found in all three types of networks (PPI, anatomy-based gene networks, and integrated networks) for zebrafish and mouse to enable a valid comparison.

### The framework to integrate anatomy ontology data with PPI networks

#### Construction of the anatomy-based gene networks

**Fig. 1:**
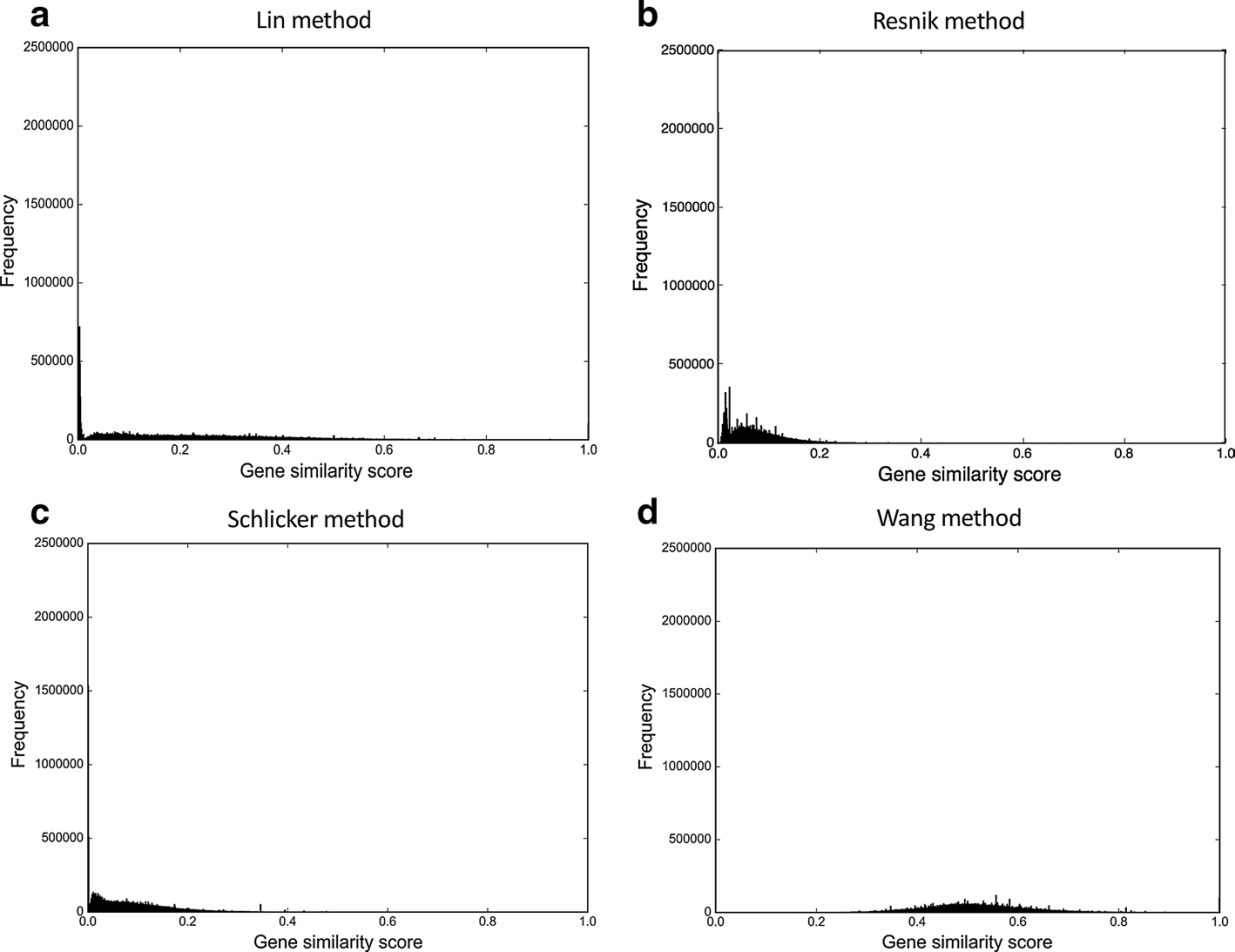
The gene similarity score distributions for the zebrafish unfiltered anatomy-based gene networks. The networks were constructed by **a** Lin method, **b** Resnik method, **c** Schlicker method, and **d** Wang method

**Fig. 2:**
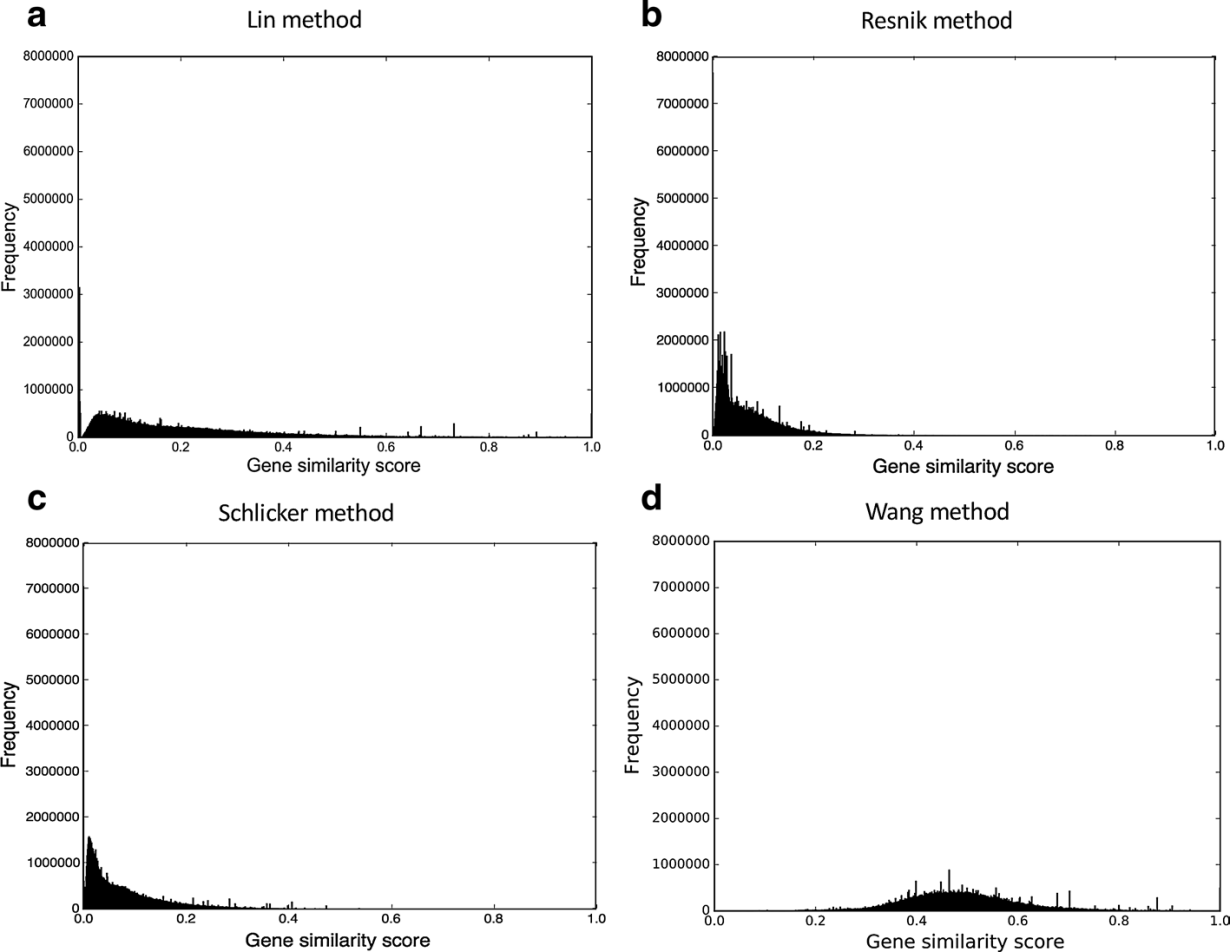
The gene similarity score distributions for the mouse unfiltered anatomy-based gene networks. The networks were constructed by **a** Lin method, **b** Resnik method, **c** Schlicker method, and **d** Wang method

When constructing the anatomy-based gene networks, we used original anatomical profiles (before the reconciliation) to retain all of their genes in the networks. We used the reconciled anatomical profiles only for the evaluation of the networks. The gene similarity score distributions for the four types of unfiltered anatomy-based gene networks (Lin, Resnik, Schlicker, and Wang) for the zebrafish and the mouse are shown in Figs. 1 and 2, respectively. The gene similarity scores for Lin, Resnik, and Schlicker methods were distributed approximately between 0 to 0.40 range. In contrast, the distribution for the Wang method was symmetrical around the 0.50 region and spanned approximately between 0.2 to 0.8 range.

Obtaining these distributions was critical to determine the gene similarity score cutoff applied to each network. For example, applying 0.7 as the cutoff for the Wang anatomy-based gene network for the zebrafish, generated a filtered network with 5386 genes and 789,282 interactions; if the same 0.7 cutoff was applied to the zebrafish Resnik network, the filtered network only had 30 genes and 31 interactions. If these two networks were evaluated, the changes in the number of genes and the number of interactions would have a significant effect on their performance. Therefore, a cutoff had to be applied to keep the network size relatively constant among different networks. However, it was difficult to practically apply cutoffs to keep the exact number of genes and interactions among the networks. Therefore, using a trial and error method, we applied different cutoffs to anatomy-based gene networks to keep the number of interactions between 500,000 and 750,000 range. The statistics for the network sizes of filtered and unfiltered anatomy-based gene networks and their cutoffs are shown in Tables 2 and 3 for zebrafish and mouse, respectively.

**Table 2:** The statistics for the unfiltered and filtered anatomy-based gene networks for zebrafish

	Lin method	Resnik method	Schlicker method	Wang method
Number of genes in the unfiltered network	5405	5405	5405	5405
Number of interactions in the unfiltered network	14,534,897	14,534,897	14,534,897	14,604,258
The gene similarity score cutoff	0.55	0.18	0.24	0.70
Number of genes in the filtered network	5387	4909	5401	5386
Number of interactions in the filtered network	700,138	712,286	692,539	789,282

**Table 3:** The statistics for the unfiltered and filtered anatomy-based gene networks for mouse

	Lin method	Resnik method	Schlicker method	Wang method
Number of genes in the unfiltered network	14,652	14,652	14,652	14,652
Number of interactions in the unfiltered network	107,094,117	107,094,117	107,094,117	107,324,905
The gene similarity score cutoff	0.9	0.32	0.41	0.95
Number of genes in the filtered network	9784	10,081	12,755	9126
Number of interactions in the filtered network	588,359	536,602	522,183	510,139

#### Integration of the anatomy-based gene networks with the STRING PPI networks

During the integration, we combined unfiltered anatomy-based gene networks for zebrafish and mouse with the corresponding STRING PPI networks. When selecting the gene similarity cutoffs for filtering the integrated networks, we considered their gene similarity score distributions, as explained in the previous section. The statistics for the filtered and unfiltered network sizes are shown in Table 4 for zebrafish and Table 5 for mouse, respectively. The generated integrated networks were larger than the anatomy-based gene networks in terms of the number of genes and the interactions. For instance, the Wang anatomy-based gene network for the zebrafish had 5405 genes and 14,604,258 interactions (Table 2), whereas the zebrafish integrated network for the Wang method had 25,375 genes and 26,821,274 interactions (Table 4). During the integration, the 5405 genes in the anatomy-based gene network were integrated with the 23,018 genes in the zebrafish PPI network, which caused an increase in the network size. The common genes and interactions were retained according to the integration formula in Eq. (5), and the genes and the interactions that were unique to one network were also included in the integrated network if the final gene similarity score was above the cutoff. Therefore, the integrated networks were more complete in terms of the number of genes and the information contained.

**Table 4:** The statistics for the unfiltered and filtered integrated networks for zebrafish

	Lin method	Resnik method	Schlicker method	Wang method
Number of genes in the unfiltered network	25,375	25,375	25,375	25,375
Number of interactions in the unfiltered network	26,753,086	26,753,086	26,753,086	26,821,274
The gene similarity score cutoff	0.33	0.23	0.24	0.4
Number of genes in the filtered network	17,394	20,066	20,929	13,940
Number of interactions in the filtered network	730,855	726,589	690,208	744,519

**Table 5:** The statistics for the unfiltered and filtered integrated networks for mouse

	Lin method	Resnik method	Schlicker method	Wang method
Number of genes in the unfiltered network	27,097	27,097	27,097	27,097
Number of interactions in the unfiltered network	111,461,010	111,461,010	111,461,010	111,690,355
The gene similarity score cutoff	0.50	0.27	0.30	0.53
Number of genes in the filtered network	13,125	17,898	18,002	12,916
Number of interactions in the filtered network	653,848	661,619	613,671	712,720

**Fig. 3:**
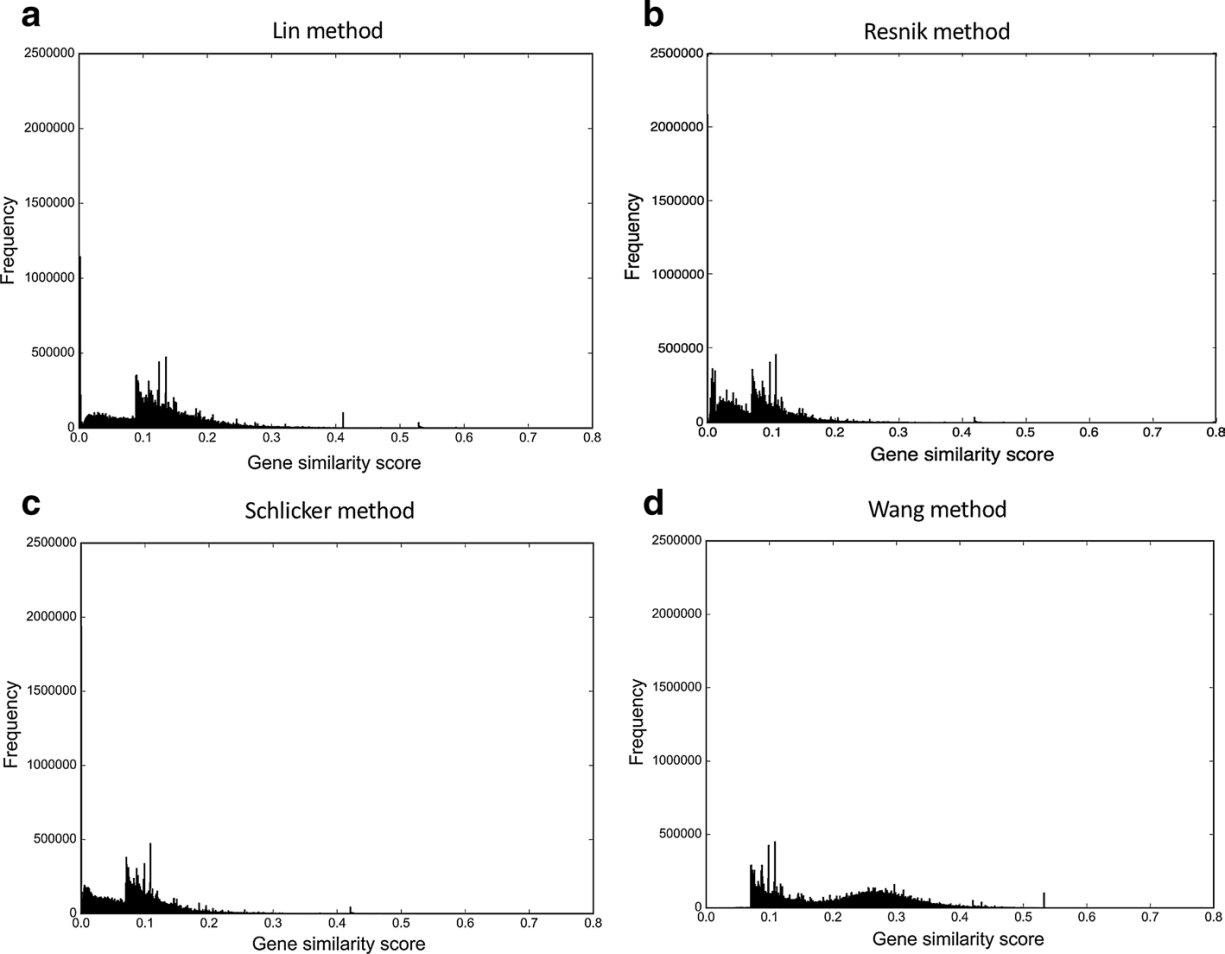
The gene similarity score distributions for the zebrafish unfiltered integrated networks. The networks were constructed by **a** Lin method, **b** Resnik method, **c** Schlicker method, and **d** Wang method

**Fig. 4:**
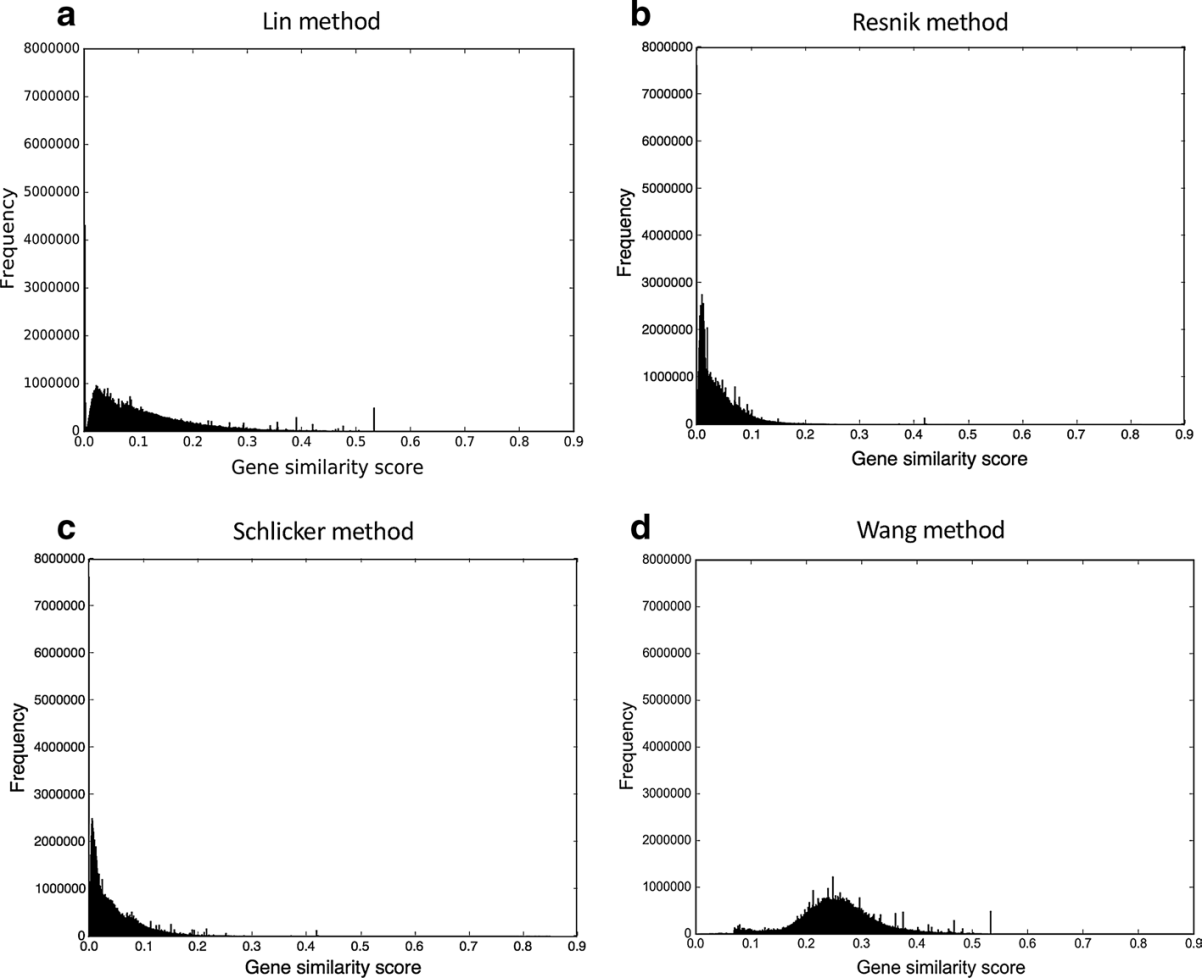
The gene similarity score distributions for the mouse unfiltered integrated networks. The networks were constructed by **a** Lin method, **b** Resnik method, **c** Schlicker method, and **d** Wang method

The gene similarity score distributions for the integrated networks for zebrafish and mouse are shown in Figs. 3 and 4, respectively. When compared to the score distributions of the corresponding anatomy-based gene networks shown in Figs. 1 and 2, the score distributions of the integrated networks were right-skewed. Especially, the gene similarity scores of the Wang integrated networks were shifted to 0–0.50 region contrary to the symmetrical distribution (around 0.5) observed for the Wang anatomy-based gene networks. This was due to the effect of the integration, because only those interactions that had high similarity scores in both anatomy-based gene networks and the PPI networks received higher scores in the integrated networks. Most of the interactions in the anatomy-based gene networks had a low support from the PPI networks, thus the gene similarity score distributions of the integrated networks were right-skewed. By applying the cutoffs shown in Tables 4 and 5, the interactions with high similarity scores that received support from both the PPI and the anatomy-based gene networks could be selected. Moreover, the integration had increased the number of interactions of individual proteins (degree) as shown in the comparisons of degree distributions between PPI and integrated networks for zebrafish and mouse in Additional file 1: Fig. S3.

### Evaluation of the candidate gene predictions

**Fig. 5:**
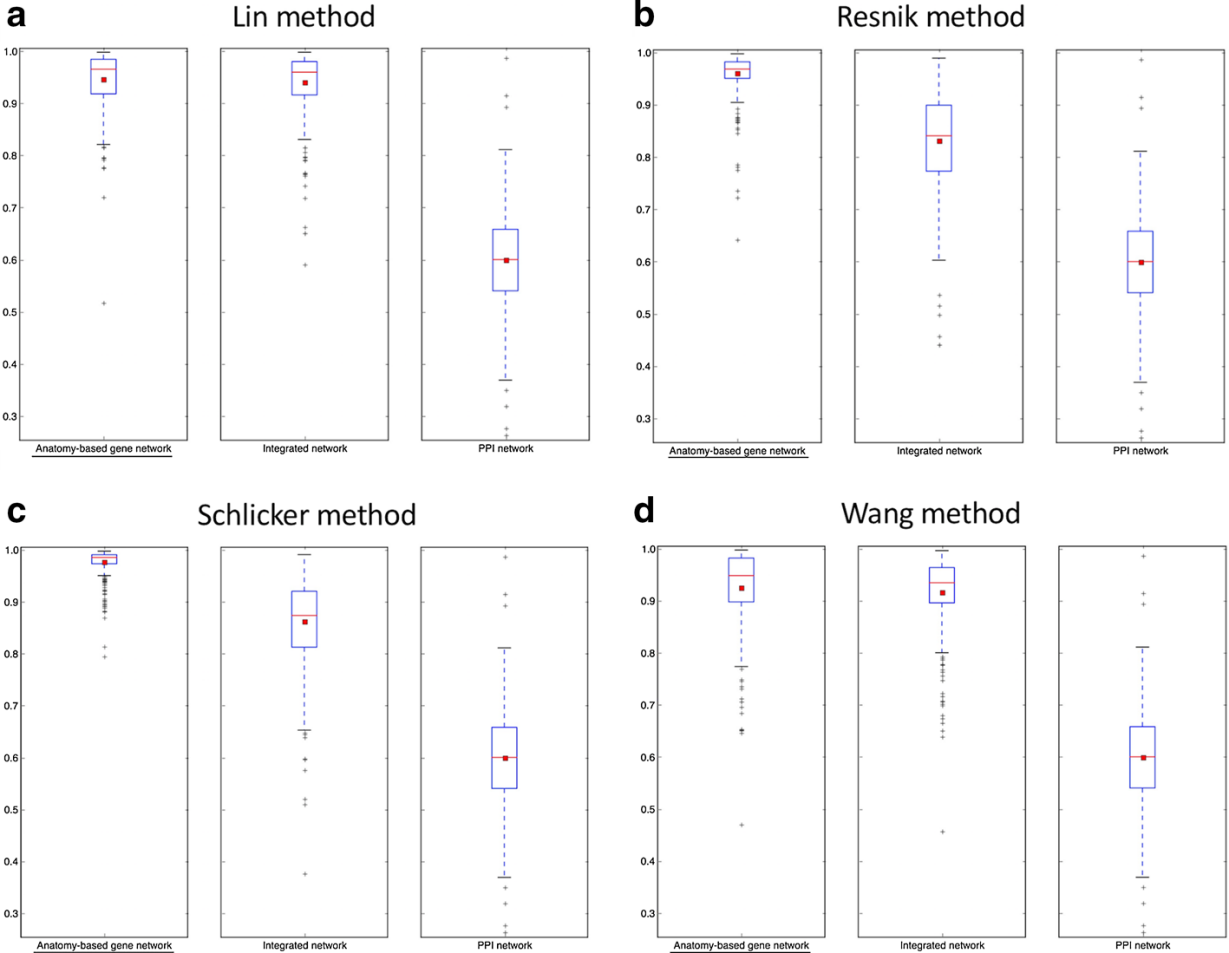
The boxplot comparisons of the AUC distributions of ROC curves for zebrafish networks. The distributions for filtered PPI networks are compared with filtered anatomy-based gene networks and integrated networks constructed by **a** Lin method, **b** Resnik method, **c** Schlicker method, and **d** Wang method. In the boxplots, the red line and the square represent the median and mean, respectively, and the name of the best performing network is underlined

**Fig. 6:**
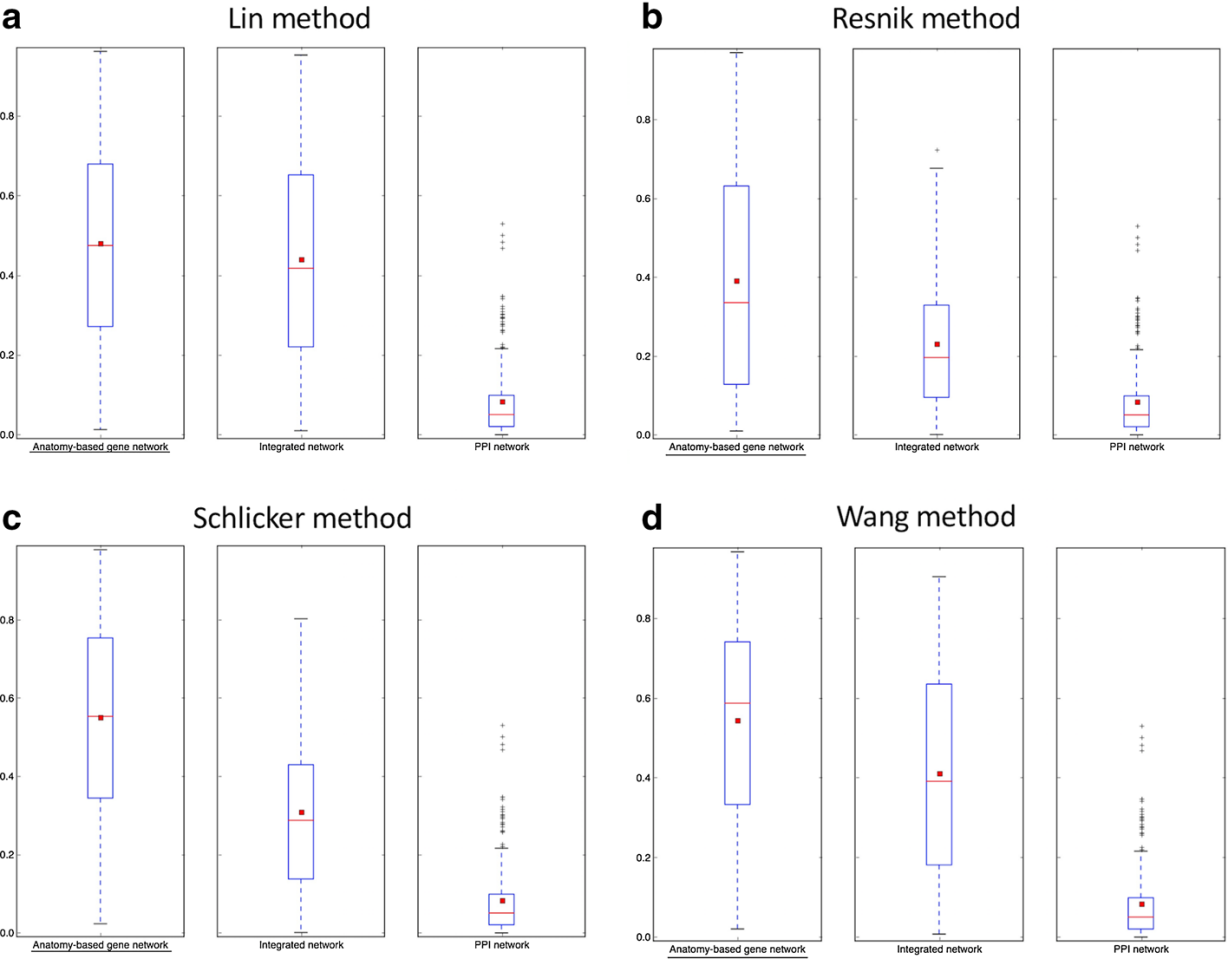
The boxplot comparisons of the AUC distributions of precision-recall curves for zebrafish networks. The distributions for filtered PPI networks are compared with filtered anatomy-based gene networks and integrated networks constructed by **a** Lin method, **b** Resnik method, **c** Schlicker method, and **d** Wang method. In the boxplots, the red line and the square represent the median and mean, respectively, and the name of the best performing network is underlined

**Fig. 7:**
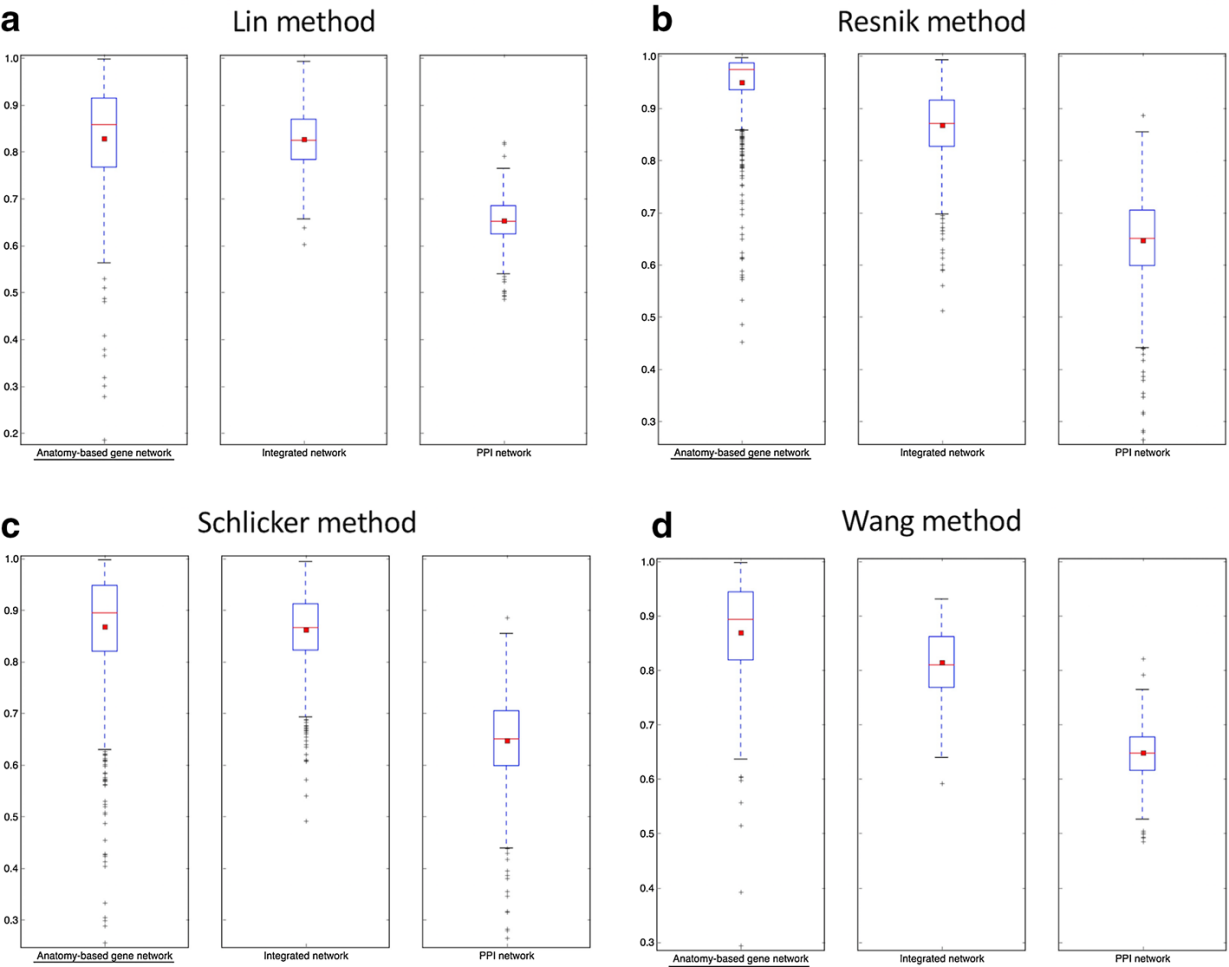
The boxplot comparisons of the AUC distributions of ROC curves for mouse networks. The distributions for filtered PPI networks are compared with filtered anatomy-based gene networks and integrated networks constructed by **a** Lin method, **b** Resnik method, **c** Schlicker method, and **d** Wang method. In the boxplots, the red line and the square represent the median and mean, respectively, and the name of the best performing network is underlined

**Fig. 8:**
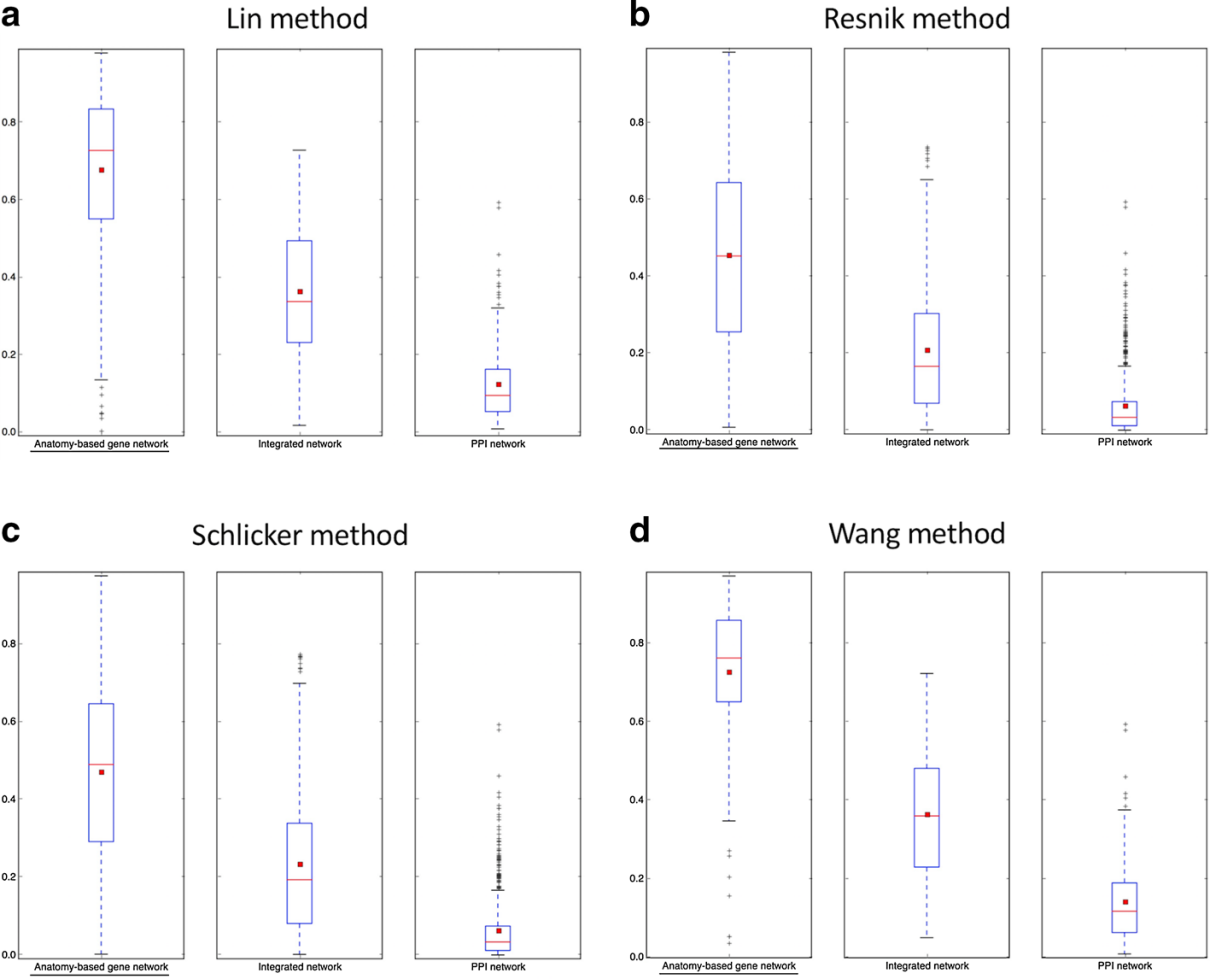
The boxplot comparisons of the AUC distributions of precision-recall curves for mouse networks. The distributions for filtered PPI networks are compared with filtered anatomy-based gene networks and integrated networks constructed by **a** Lin method, **b** Resnik method, **c** Schlicker method, and **d** Wang method. In the boxplots, the red line and the square represent the median and mean, respectively, and the name of the best performing network is underlined

The purpose of integrating anatomy ontology data with PPI networks was to improve the accuracy of predicting candidate genes for anatomical entities. The boxplot comparisons of the AUC distributions of ROC and precision-recall curves for zebrafish networks are given in Figs. 5 and 6, respectively. The boxplot comparisons of the AUC distributions of ROC and precision-recall curves for mouse networks are given in Figs. 7 and 8, respectively. Each figure compares the AUC distributions for three network types: anatomy-based gene networks, integrated networks, and PPI networks for the four semantic similarity calculation methods.

According to the boxplot comparisons, the AUC distributions of ROC and precision-recall curves for the integrated networks were higher than those of the PPI networks for both zebrafish and mouse for all four semantic similarity calculation methods. This confirmed that integration of anatomy ontology data improved the candidate gene prediction accuracy for anatomical entities compared to the PPI networks for the two model organisms. The differences in the network performances for the four semantic similarity methods could have been due to the difference in the way each method captured the semantic information and the effect of the network cutoffs used for different networks. Although anatomy-based gene networks outperformed integrated networks in the boxplot comparisons, integrated networks were more suitable for candidate gene prediction for anatomical entities in practice. For instance, anatomy-based gene networks were much smaller (incomplete) than integrated networks, thus the genes that could have been predicted using anatomy-based gene networks were very limited. Moreover, integrated networks included support from multiple data sources such as anatomical annotations and other molecular interactions, which could have improved their prediction power.

### Further validation of the prediction results

The further validations of the prediction results were performed using anatomy-based and integrated networks constructed using the Wang method. We made this selection because of the symmetric gene similarity score distributions (Figs. 1 and 2) and annotation-independent characteristics of the Wang anatomy-based gene networks. The score distributions of anatomy-based gene networks for the other semantic similarity methods were right-skewed (majority of similarity scores were at 0–0.1 region) and annotation-dependent. Therefore, the Wang semantic similarity calculation method generated the best networks for further validation of the prediction results.

**Fig. 9:**
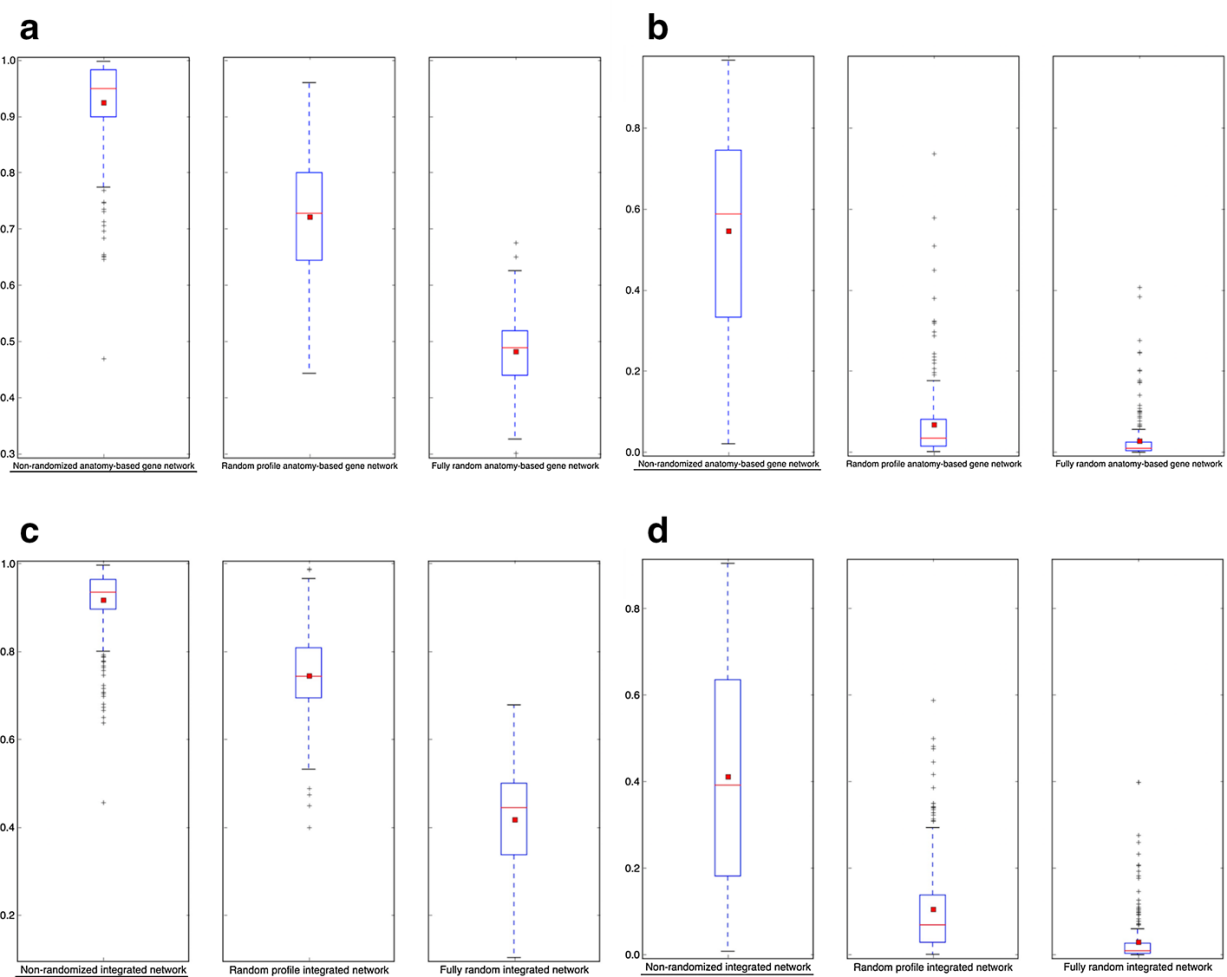
The network performance comparisons between non-randomized and randomized networks for zebrafish. The boxplot comparisons of the AUC distributions for **a** ROC and **b** precision–recall curves for the filtered non-randomized anatomy-based gene network, randomized profile anatomy-based gene network, and fully randomized anatomy-based gene network for the Wang method for the zebrafish. The boxplot comparisons of the AUC distributions for **c** ROC and **d** precision–recall curves for the filtered non-randomized integrated network, randomized profile integrated network, and fully randomized integrated network for the Wang method for the zebrafish. In the boxplots, the red line and the square represent the median and mean, respectively, and the name of the best performing network is underlined

The AUC distribution comparisons of ROC and precision-recall curves for non-randomized anatomy-based gene networks and integrated networks for the Wang method with their fully randomized and randomized profile counterparts for zebrafish and mouse are shown in Fig. 9 and Additional file 1: Fig. S4, respectively. According to the comparisons, non-randomized networks showed better performances (higher AUC score distributions) compared to the randomized networks in both network types. When comparing the two randomization methods, randomized profile networks, which were constructed by only randomizing the anatomical profiles, showed higher performances than the fully randomized networks. These comparisons with randomized networks indicated that the higher candidate gene prediction performances of the anatomy-based gene networks and the integrated networks were due to their biological significance.

**Fig. 10:**
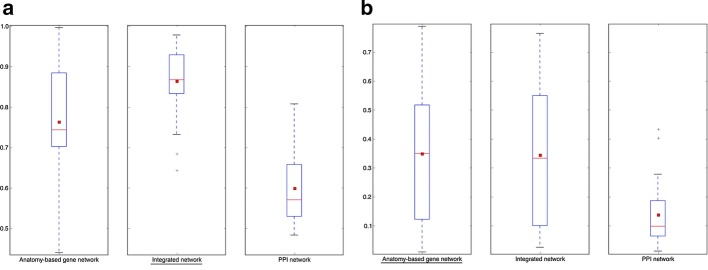
The network performance comparisons for zebrafish networks when evaluated by randomly removed 30 anatomical entities. The boxplot comparisons of the AUC distributions for **a** ROC and **b** precision–recall curves for the filtered integrated network, PPI network, and anatomy-based gene network for the Wang method for zebrafish. The integrated network and the anatomy-based gene network were generated using the zebrafish anatomy profiles after randomly removing 30 anatomical entities, which had at least 10 gene annotations. The same 30 entities were used for the evaluation. In the boxplots, the red line and the square represent the median and mean, respectively, and the name of the best performing network is underlined

To test the effect of the circular use of the same anatomical profiles for network construction and evaluation, the AUC distribution comparisons of ROC and precision-recall curves for Wang anatomy-based and integrated networks evaluated using 30 anatomical entities that were not used for the construction of the networks for zebrafish and mouse are given in Fig. 10 and Additional file 1: Fig. S5, respectively. According to these figures, the integrated networks performed better than the PPI networks and the anatomy-based gene networks, when evaluated using the 30 anatomical entities that were not used for the network construction. Here, the 30 anatomical entities were selected as a representative number, and we repeated the experiment with 10, 60, 100, and 150 anatomical entities randomly removed at a time for zebrafish and observed similar results (Additional file 1: Figs. S6 and S7). However, the removal of a higher number of anatomical entities could have reduced the anatomical information captured by the semantic networks. On the other hand, the removal of a low number of anatomical entities could have decreased the number of entities used for the evaluation. Therefore, the 30 anatomical entities were considered to reduce the limitations of removal of too few or too many entities during the evaluation.

**Fig. 11:**
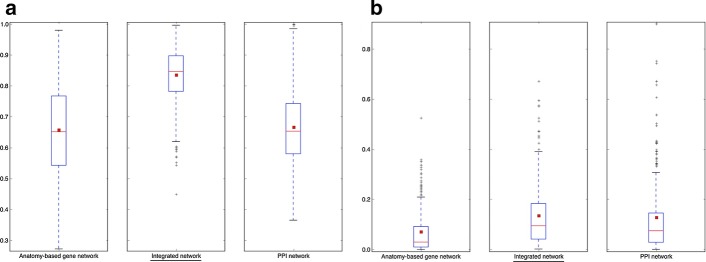
The network performance comparisons for zebrafish networks when evaluated by Gene Ontology-Biological Process (GO-BP) entities. The boxplot comparisons of the AUC distributions of **a** ROC and **b** precision–recall curves for the filtered integrated network, PPI network, and anatomy-based gene network for the Wang method in zebrafish. The networks were evaluated using the annotation profiles containing GO-BP entities for the zebrafish genes. In the boxplots, the red line and the square represent the median and mean, respectively, and the name of the best performing network is underlined

The AUC distribution comparisons of ROC and precision-recall curves for the Wang anatomy-based and integrated networks and the PPI networks evaluated using GO-BP profiles for the zebrafish and mouse are shown in Fig. 11 and Additional file 1: Fig. S8, respectively. Because the anatomy-based gene networks and the integrated networks were constructed using the zebrafish and mouse anatomy profiles, the GO-BP annotations, which were used for the evaluation, did not have a direct influence on the network construction. According to the results, the integrated networks performed better than both the PPI and anatomy-based gene networks, when evaluated by the GO-BP profiles.

## Discussion

**Fig. 12:**
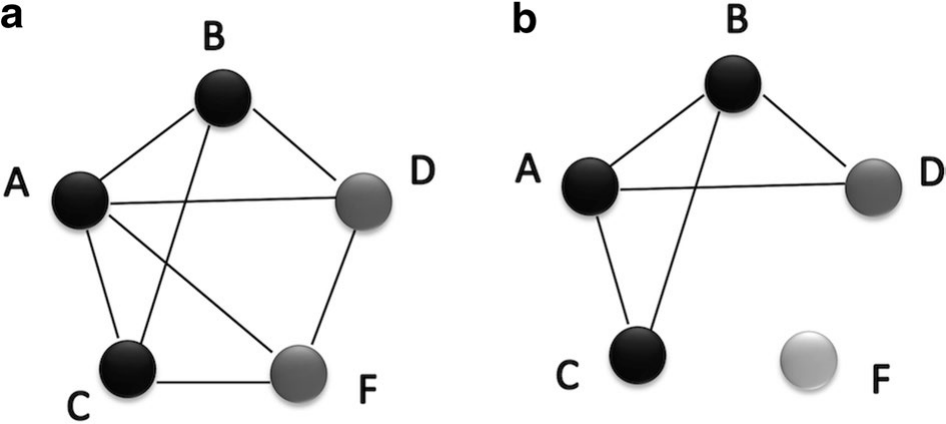
A hypothetical representation showing how the network integration filters false positive interactions. This scenario compares candidate gene predictions between a **a** PPI network and an **b** anatomy-based gene network. The nodes A, B, and C (colored in black) in both networks represent three genes known to be associated with a certain anatomical entity denoted as *entity 1*. In the PPI network (**a**), genes D and F are predicted to be associated with *entity 1* because genes D and F interact with genes A, B, and C that are known to be associated with the *entity 1*. In contrast, the anatomy-based gene network (**b**) only predicts D as a potential candidate for *entity 1* because the gene F does not have any interaction with other genes annotated with *entity 1*. The absence of interactions of gene F in gene network (**b**) can be due to two reasons: (1) it is not annotated with any anatomical entities, (2) it is not annotated with entities that are similar to the anatomy entities associated with genes A, B, or C. The anatomy-based gene network (**b**) is built entirely on anatomy ontology information, thus it provides a different interaction structure. Hypothetically, the gene F could have formed false positive interactions in the PPI network, and the integrative use of the anatomy-based gene network may reduce the false positives by filtering them

The goal of this work was to test whether the integration of anatomy ontology relationships with PPI networks enhanced the network-based candidate gene prediction accuracy when predicting gene candidates associated with anatomical entities. According to the network performance evaluations (Figs. 5, 6, 7 and 8), the integrated networks performed better than the PPI networks. Here, we assumed that the increased candidate gene prediction accuracy was due to the increased network quality. In the integrated networks, the gene interactions that received high interaction scores were the ones that received a higher support from both the PPI and anatomy-based gene networks. This filtered out false positive interactions (Fig. 12) in the PPI networks if those interactions were not supported by the anatomy-based gene networks, that is, if they received low scores from the anatomy-based gene networks. On the other hand, gene interactions with low scores in the PPI networks were enhanced if the interactions were supported by the anatomy-based gene networks, that is, if they had high similarity scores in the anatomy-based gene networks. In cases where the gene similarity score was zero in anatomy-based gene networks due to lack of anatomical term annotations, the support for those interactions from the PPI networks should have been extremely high for those interactions to be retained in the integrated networks. Similarly, if two proteins were not interacting in a PPI network, they needed a very high support from the anatomy-based gene network to be retained in the integrated network. Furthermore, a protein usually interacts with multiple proteins, thus a high quality network should have more proteins with large degrees [16]. The integrated networks not only increased the network size, but also increased the average degrees of proteins in the network, which improved the completeness and the quality (Additional file 1: Fig. S3). These improved network characteristics in the integrated networks, i.e., the improvements in network quality, increased the candidate gene prediction performances of the networks.

The robustness of the integrated networks for improved candidate gene predictions was further demonstrated by performing evaluations under different settings. For example, we used both ROC curves and precision-recall curves for the evaluations, because they had been extensively used for evaluating network-based candidate gene predictions in the literature [16, 19, 40, 61]. In majority of previous candidate gene prediction studies, only one or few functions were selected at a time and their ROC and precision-recall curves were compared directly in graphs during the performance evaluations [16, 19, 20]. Here, instead of plotting curve comparisons for few anatomical entities, we used boxplot distributions of the AUC values of these curves to increase the number of anatomical entities used for network performance evaluations. The proteins responsible for regulation of a specific molecular function or an anatomical entity are usually clustered together forming functional modules [16, 62]. If only few anatomical entities were used for the evaluation by comparing their ROC and precision-recall curves, only the protein modules responsible for the selected anatomical entities would be evaluated. In this work, it was required to evaluate the entire network structure, not limiting to a small portion of the network, to completely assess the effect of the network integration. Therefore, we used the maximum number of possible anatomical entities for the evaluation by comparing their AUC values of ROC and precision-recall curves using boxplots. Moreover, we used four semantic similarity methods (Lin, Resnik, Schlicker, and Wang), which have been widely used to calculate semantic similarity [34, 38, 39] using ontology information, to generate semantic networks for the two model organisms (zebrafish and mouse). Under all these experimental settings, the integrated networks performed better than the PPI networks, strengthening the conclusion that the integration of the gene-anatomical entity associations from literature with the PPI networks increased the candidate gene prediction accuracy for anatomical entities.

The results from the network predictions compared to those obtained using the random networks (Fig. 9 and Additional file 1: Fig. S4) demonstrated that the higher candidate gene prediction performance observed in the integrated networks had biological significance and were not due to random error/chance. Of the two randomization procedures used, the randomized profile networks, which were generated by only randomizing the anatomical profiles, performed better than the fully randomized networks that were constructed by completely randomizing the entire networks. When only the anatomical profiles were randomized, the original number of annotations per gene was kept constant even after the randomization, which may have reduced the randomization effect by including closely related anatomical entities for the same gene. This may explain their better performance compared to the fully randomized networks.

A concern in the analysis was in regard to the potentially circular use of the same anatomical profiles for the construction and the evaluation of the networks (anatomy-based gene networks and integrated networks). The results of the two experiments designed to investigate the effect of the circular use in zebrafish and mouse: (1) evaluation using 30 anatomical entities that were removed before the integrated network construction (Fig. 10 and Additional file 1: Fig. S5) and (2) evaluation using the GO-BP profiles (Fig. 11 and Additional file 1: Fig. S8), indicated that integrated networks had better performances than both anatomy-based and PPI networks. In both experiments, the annotations used for the network construction were not used for the evaluation, which meant that the increased performances observed in the integrated networks were not due to the circular use of the same anatomical profiles. Previous studies [20, 38, 39] that used semantic networks for candidate gene predictions have been using networks that were directly based on the ontology. Our results showed the integration of semantic networks with PPI networks as a better option to remove the bias of the predictions. Furthermore, anatomy-based gene networks contained a limited number of genes because only the genes with anatomy ontology annotations were included, thus they were not practical for candidate gene prediction as the integrated networks. For example, the zebrafish filtered anatomy-based gene network constructed using the Schlicker method contained 5401 genes (Table 2), whereas the corresponding integrated network contained 20,929 genes (Table 4). Integration with PPI network data have added new genes without anatomical annotations to the integrated networks, many of which could be novel candidates for anatomical entities. Moreover, the anatomy-based gene networks represent the gene organization in the network using only their anatomical annotations, while the PPI networks include many molecular interactions supported by experiments. Integration of these two different types of networks combines different biological knowledge; thus, the integrated networks are better suited for candidate gene predictions.

In literature, there have been several occasions where semantic networks were integrated with PPI networks for candidate gene prediction [19, 40, 63, 64]. Our results further validated that the integrated networks outperform the pure semantic networks. In previous studies including the most recent ones [19, 64], semantic networks have been mainly used for predicting disease genes or genes associated with GO entities. To our knowledge, this is the first work that uses semantic networks for predicting anatomical entities. There is a pressing need for accurate methods to predict candidate genes associated with anatomical entities as only few genes were annotated to the majority of anatomical entities, such as the pelvic fin (15 genes in the zebrafish anatomical profiles). Discovering new genes associated with anatomical entities leads to a clearer understanding of the molecular mechanisms underlying anatomical development. Moreover, such discoveries support the progress of emerging evolutionary developmental biology studies [2, 65]. Therefore, our work significantly contributes to the progress of developmental biology.

One of the future directions of this work is to analyze protein module changes associated with the fin to limb transition [4, 66] using the integrated networks generated in this study. We are interested in identifying protein modules related to pectoral and pelvic fin in zebrafish and comparing them with corresponding forelimb and hindlimb modules in mouse to study PPI modular changes associated with the fin to limb transition. The reason for selecting zebrafish and mouse for this study was to support this module comparison, which may unravel useful knowledge regarding an important phenotypic transition [4, 66] in evolutionary biology. Because the modules are to be identified computationally, a better network quality is essential. As we confirmed in this study, the integrated networks provide more accurate candidate gene predictions than conventional PPI networks, thus can have broader applications in developmental biology.

Another interesting future challenge is to include quality terms using Phenotype and Trait Ontology to analyze the genes associated with certain qualities of anatomical entities, such as the size or the presence and absence of an anatomical term [67]. For this task, a computational framework must be established to include composite entity-quality terms. Alternatively, phenotype ontologies, which already include quality of an anatomical entity, such as the Human Phenotype Ontology [68] and the Mammalian Phenotype Ontology [69], can be directly used for the integrative framework.

This integrative approach will be extremely useful for studying disease phenotypes in humans and other model organisms. Predicting disease genes using biological networks is widespread due to the associated medical implications [18, 19, 70, 71], and the challenge is to improve the accuracy of the predictions. Using this integrative method, experimental knowledge regarding known gene-disease associations can be integrated with human gene/PPI networks. For this purpose, Human Disease Ontology [72, 73] can be used instead the Uberon to semantically capture the gene-disease annotations. Therefore, the integrative framework used in this work is adaptable to a broad number of research questions and is a powerful tool for the bioinformatics community.

The major limitation of our integrative approach is its reliance on current gene-anatomical entity associations in literature. The completeness of these associations varies on the model organism. For instance, some model organisms, such as human and mouse, contain more associations than others because more studies have been focused on them. For instance, as shown in Table 1, mouse anatomical profiles used for this work contain a higher number of genes with known annotations (14,652) than zebrafish (5405). More complete and accurate associations improve the quality of the anatomy-based gene networks. For model organisms with few associations and non-model organisms without any associations, this integrative method is inefficient.

There is a lack of bioinformatics tools and code to address large-scale network integration problems, and built-in codes and libraries for semantic similarity calculations are not readily available for the Python programming language [58]. Therefore, the Python scripts written for this research will be extended to a Python library focused on large-scale network integration and construction of semantic networks using semantic similarity calculation methods.

## Conclusions

This work focused on improving the candidate gene prediction accuracy for anatomical entities using the PPI networks by integrating known gene-anatomical entity knowledge via anatomy ontology data. According to candidate gene prediction performances evaluated under different computational settings (four semantic similarity calculation methods: Lin, Resnik, Schlicker, and Wang; two model organisms: zebrafish and mouse; two evaluation curve types: ROC and precision-recall curves), the integrated networks outperformed PPI networks and were better for predicting candidate genes for anatomical entities. Furthermore, the integrated networks proved better than either anatomy-based or PPI networks. Our study showed that the integration of the experimental knowledge via anatomy ontology increases candidate gene prediction accuracy for anatomical entities and provided a computational platform to better future developmental biology studies.

## Methods

### Data sources

We retrieved the PPI networks for zebrafish and mouse from the STRING database (01/05/2018; version 10.5) [45]. The proteins in the PPI networks were represented by unique STRING IDs, and we replaced them with corresponding gene names/symbols using the STRING ID to gene name mappings from the meta-data retrieved from the STRING database (01/05/2018) to facilitate network integration in later stages. Usually, raw PPI networks are too large for downstream analyses. Therefore, we filtered the networks based on the recommended 0.7 gene interaction/combined score cutoff [21].

To construct anatomy-based gene networks, initially, anatomical profiles had to be constructed. An anatomical profile represents the multiple anatomical entity annotations for a gene. We obtained known gene-anatomical entity relationships for zebrafish and mouse from the Monarch Initiative repository (01/08/2018) [29, 30]. The Monarch Initiative retrieves genes and their anatomical entity annotations for zebrafish and mouse from the zebrafish [46] and mouse [47] model organism databases, respectively and associates them with the corresponding Uberon [33] anatomical entities. The annotations available in the Monarch Initiative were pre-processed and cross-checked with other model organism annotations to remove uncertain gene-anatomical entity associations that may result when the expression of multiple genes were simultaneously disrupted to observe the effect on a given phenotype [29]. The Uberon anatomy ontology used by the Monarch Initiative is a cross-species ontology, which integrates species-specific anatomy ontologies, such as Mouse Anatomy Ontology and Zebrafish Anatomy Ontology, in a species-neutral way [31–33]. This multi-species integration makes Uberon a prime candidate for large-scale computational analyses, involving multiple organisms.

### The framework to integrate anatomy ontology data with PPI networks

#### Construction of the anatomy-based gene networks

To construct anatomy-based gene networks, we arranged the gene-anatomical entity associations into the following format where *G*_*1*_ and *G*_*2*_ represent two genes, and (*t*_*a1*_,* t*_*a2*_*… t*_*am*_) and (*t*_*b1*_,* t*_*b2*_*…t*_*bn*_) represent their associated anatomical entities (Uberon entities), respectively:

$$\begin{gathered} G_{1} : \, \left( {t_{a1} , \, t_{a2} ... \, t_{am} } \right), \hfill \\ G_{2} : \, \left( {t_{b1} , \, t_{b2} ...t_{bn} } \right). \hfill \\ \end{gathered}$$

(As an example, the zebrafish anatomical profiles are available in Additional file 2). Then, we calculated semantic similarity scores between anatomical entities annotated to gene pairs, and we aggregated these scores to calculate the gene similarity scores between all gene pairs (Additional file 1: Fig. S1). We used four methods to calculate semantic similarity between Uberon terms: Wang method [48], Resnik method [49], Lin method [50], and Schlicker method [51]. These four methods were selected because they have been widely used for constructing semantic networks [34, 38, 39]. The equations and definitions for these methods are given in Additional file 3. The latter three methods are based on calculating the information content (IC) of each term in the ontology hierarchy. The IC measures how specific and informative a specified term is based on its probability of occurrence in a given corpus, such as the Uberon ontology [49, 52]. The IC of a term increases with its specificity in the corpus. The Wang method does not use the IC [48]. It only depends on the ontology structure and the relationships between the entities.

After obtaining the semantic similarities between Uberon terms using these four methods, we calculated the similarity between gene pairs using the method explained below. For instance, if the gene *G*_*1*_ is annotated with the anatomical entities: (*t*_*a1*_,* t*_*a2*_*… t*_*am*_), and the gene *G*_*2*_ is annotated with the anatomical entities: (*t*_*b1*_*, t*_*b2*_*…t*_*bn*_), then the similarity between the two genes, *sim* (*G*_*1*_,* G*_*2*_), was calculated using Eq. (1).

1 $$sim\left( {G_{1} ,G_{2} } \right) = \frac{{\mathop \sum \nolimits_{1 \le i \le m} sim\left( {t_{ai} , t(G_{2} )} \right) + \mathop \sum \nolimits_{1 \le j \le n} sim\left( {t_{bj} ,t(G_{1} )} \right)}}{m + n}$$

The ${\text{t(G}}_{{1}} {)}$ = (*t*_*a1*_,* t*_*a2*_*… t*_*am*_) and ${\text{t(G}}_{{2}} {)}$ = (*t*_*b1*_,* t*_*b2*_*…t*_*bn*_) represent anatomical profiles for gene *G*_*1*_ and gene *G*_*2*_, respectively, and the ${\text{ sim}}\left( {{\text{t}}_{{{\text{ai}}}} {\text{, t(G}}_{{2}} {)}} \right)$ represents the maximum semantic similarity between term ${\text{t}}_{{{\text{ai}}}}$ and any of the entities in ${\text{ t(G}}_{{2}} {)}$, which was calculated using Eq. (2) below.

2 $$sim\left( {t_{ai} , t(G_{2} )} \right) = \mathop {\max }\limits_{{t_{b} \in t(G_{2} )}} sim(t_{ai} , t_{b} )$$

Using Eq. (1), we calculated a similarity score for each gene pair in the anatomical profiles for zebrafish and mouse, which generated a pairwise gene similarity matrix for each semantic similarity calculation method. After obtaining a gene similarity matrix, the final step of the anatomy-based gene network construction was to connect each pair of genes with an edge. We applied a gene similarity cutoff for this purpose. If the pairwise gene similarity score between two genes was higher than the cutoff, an edge was placed to connect the two genes, otherwise, they were not connected (filtered network). We obtained suitable cutoffs for anatomy-based gene networks by analyzing their similarity score distributions, which were selected to keep the number of interactions/gene pairs approximately similar to that of the STRING PPI networks with the 0.7 cutoff for each model organism.

#### Integration of the anatomy-based gene networks with the STRING PPI networks

We performed the network integration using an accuracy-based weighting method [53] that uses the gene similarity scores for each gene interaction from the STRING PPI networks and the anatomy-based gene networks. We expected the integration to improve the candidate gene prediction accuracy for anatomical entities by improving the quality of the PPI networks. The integration assigned higher weights to the gene interactions that were supported by both PPI and anatomy-based gene networks, which improved the network quality by reducing the false positive and false negative interactions (Fig. 12).

Initially, we evaluated the PPI and anatomy-based gene networks separately using the evaluation workflow described in the next section, which was used to decide the accuracy weights for the integration of gene similarity scores. For instance, if the accuracy values for the PPI and anatomy-based gene networks were *AC*_*1*_ and *AC*_*2*_ respectively, the weights for the PPI network (*W*_*1*_) and the anatomy-based gene network (*W*_*2*_) were calculated using Eqs. (3) and (4), respectively.

3 $$W_{1} = \frac{{AC_{1} }}{{AC_{1} + AC_{2} }}$$

4 $$W_{2} = \frac{{AC_{2} }}{{AC_{1} + AC_{2} }}$$

Then, we used the weights to calculate the gene similarity scores for the integrated network, based on the gene similarity scores of the original two networks. For instance, consider the similarity between the two genes: *G*_*a*_ and *G*_*b*_. If the similarity scores from the PPI network and the anatomy-based gene network for these two genes were given by *sim*_*1*_(*G*_*a*_*, G*_*b*_) and *sim*_*2*_(*G*_*a*_,* G*_*b*_), respectively, the similarity score *sim*_*3*_(*G*_*a*_,* G*_*b*_) in the integrated network was calculated by Eq. (5) below.

5 $$sim_{3} \left( {G_{a} , G_{b} } \right) = W_{1} sim_{1} \left( {G_{a} , G_{b} } \right) + W_{2} sim_{2} \left( {G_{a} , G_{b} } \right)$$

If an interaction was not found in an original network, the similarity score was zero for that interaction; for instance, if *G*_*a*_ and *G*_*b*_ were not interacting in the PPI network, *sim*_*1*_(*G*_*a*_,* G*_*b*_) was assigned zero. During the calculation of weights, the more accurate original network obtained a higher weight and the integrated network was weighted towards the more accurate network. We used this method to integrate the zebrafish and mouse PPI networks with the four anatomy-based gene networks (Lin, Resnik, Schlicker, and Wang), which resulted in four integrated networks for each organism.

### Evaluation of the network-based candidate gene prediction

For the evaluation, gene name/symbol reconciliation was required to align PPI networks from STRING to anatomical profiles retrieved from the Monarch Initiative repository. The STRING database obtains data from various data sources, such as Entrez Gene database [54] and UniProt knowledgebase [55], whereas the Monarch Initiative repository obtains data from model organism databases. Occasionally, the gene names do not match. Therefore, we computationally reconciled gene names of the two data sources using three steps: (1) matching the genes directly using their names/symbols, (2) matching the genes using their Ensembl identifiers, and (3) matching the remaining gene names in the anatomical profiles to the synonyms available in the STRING database (Ensembl identifier and synonym matching were performed using meta-data retrieved from the STRING database; 01/05/2018). Each step attempted to sequentially minimize the number of gene mismatches.

Then, we used these reconciled zebrafish and mouse anatomical profiles for the evaluation. We filtered the profiles first to only keep the anatomical entities that contained at least 10 gene annotations. We then used leave-one-out cross-validation on one anatomical entity at a time and generated a ROC curve and a precision-recall curve for each entity. Here, out of all the genes annotated to the anatomical entity of interest, the association of one gene was assumed to be unknown at a time, and the other genes were used to predict the anatomical entity of that unknown gene. This process was repeated until all the genes were selected for anatomical entity prediction. Finally, we compared the distribution of AUC values for all the anatomical entities for the three types of zebrafish and mouse networks (integrated networks, anatomy-based gene networks, and PPI networks) for each semantic similarity method (Lin, Resnik, Schlicker, and Wang). We used the Hishigaki method [16, 56] as the network-based candidate gene prediction algorithm for which Eq. (6) is given below.

6 $$prediction\,score = \frac{{\left( {n_{f\left( u \right)} - e_{f} } \right)^{2} }}{{e_{f} }}$$

In Eq. (6), ${\text{n}}_{{\text{f(u)}}}$ denotes the number of genes with the considered anatomical term (*f*) in the neighborhood of the gene of interest (*u*). Generally, the length of the neighborhood can be defined by the user but the immediate neighborhood (a length of one edge from the gene *u*) is shown to yield better results [56]; therefore, we only considered the immediate neighborhood of a gene for predictions. The expected frequency for the anatomical entity is given by ${\text{e}}_{{\text{f}}}$, which was calculated according to Eq. (7) below.

7 $$e_{f} = \frac{{tot_{f} *n\left( u \right)}}{{tot_{N} }}$$

In Eq. (7), ${\text{tot}}_{{\text{f}}}$ denotes the total number of genes annotated with the given anatomical term (*f*) in the network and ${\text{tot}}_{{\text{N}}}$ indicates the total number of genes in the network. The total number of genes in the immediate neighborhood of the gene of interest (*u*) is denoted by *n*(*u*).

### Further validation of the prediction results

It was important to understand the biological significance of the candidate gene prediction results. For instance, if the integrated networks performed better than the PPI networks, it needed to be confirmed that the increased performance was due to the biological significance of integrating experimental anatomical data via anatomy ontology annotations and not due to random error/noise. For this purpose, we generated fully randomized networks with the same number of nodes and the same number of edges as those in the integrated and anatomy-based gene networks constructed using the Wang method for zebrafish and mouse. Furthermore, we generated another type of a randomized network by only randomizing the reconciled anatomical profiles by randomly assigning anatomical entities to each gene to match the original number of annotations, and then, constructing the anatomy-based gene networks and integrated networks using the Wang method. The second method was only a partial randomization because only the anatomical profiles were randomized, and the number of interactions were different from the original networks. From herein, we call the first randomized network type as ‘fully randomized networks’, and the second type as ‘randomized profile networks’. We compared the candidate gene prediction performances of the anatomy-based gene network and the integrated network constructed using the Wang method with their corresponding fully randomized and randomized profile networks for zebrafish and mouse.

A potential concern with the proposed integrative method was the usage of the same anatomical profiles for the integrated network constructions and evaluations. Therefore, if the integrated networks outperformed the PPI networks, this may have been caused by the circular use of the same anatomical profiles for the network construction and the evaluation. To further investigate this issue, we conducted two experiments. First, we randomly removed 30 anatomical entities with at least 10 gene annotations from the reconciled zebrafish and mouse anatomical profiles and constructed the anatomy-based and integrated networks from the remaining anatomical entities using the Wang method. Then, we compared the network-based candidate gene prediction performance of these networks with the zebrafish and mouse PPI networks using the removed 30 anatomical entities for the evaluation. If the performance increases in the integrated networks were only due to re-using the same anatomical entities for the evaluation, the integrated network should have not shown a performance increase compared to the PPI networks when those 30 anatomical entities were used for the evaluation because they were not involved in the network construction.

For the second experiment, we downloaded zebrafish and mouse GO annotations from the GO consortium (01/30/2018) [57] and pre-processed them to keep only the GO-BP annotations. Then, we constructed GO-BP profiles for zebrafish and mouse genes and reconciled them to only keep the genes that were found in zebrafish and mouse networks (PPI, anatomy-based gene networks, and integrated networks). Finally, we evaluated the network-based candidate gene prediction performance of the semantic networks (anatomy-based and integrated networks) constructed using the Wang method and the PPI networks for zebrafish and mouse using the reconciled GO-BP profiles. Here, the evaluation was performed by GO-BP profiles, which were not used for the construction of the anatomy-based and integrated networks.

### Implementation of the integrative framework

We implemented this integrative framework using the Python 2.7 environment [58]. All the scripts that were used for constructing the anatomy-based gene networks, integration, evaluation and further validation are available at https://doi.org/10.5281/zenodo.3470875. We generalized the scripts so that they can be used with any PPI network retrieved from the STRING database, not only limiting to mouse and zebrafish.

## Supplementary information

**Additional file 1:** This PDF file contains the supplementary figures referred throughout the manuscript.

**Additional file 2:** This text file (TXT) contains anatomical profiles for the zebrafish used for anatomy-based gene network construction.

**Additional file 3:** This PDF file contains the definitions of the four semantic similarity calculation methods (Wang method, Resnik method, Lin method, and Schlicker method) used for anatomy-based gene network and integrated network construction.

## Data Availability

The network files and the anatomy profiles used for the candidate gene predictions are available at https://doi.org/10.6084/m9.figshare.9973703.v2 and the Python scripts used for this analysis are available at https://doi.org/10.5281/zenodo.3470875.

## Abbreviations

PPI: Protein–protein interaction
GO: Gene ontology
GO-BP: Gene Ontology-Biological Process
IC: Information content
ROC: Receiver operating characteristic
AUC: Area under the curve
