# Supplementary material for: Integration of anatomy ontology data with protein–protein interaction networks improves the candidate gene prediction accuracy for anatomical entities
Source: BMC Bioinformatics. 2020 Oct 7;21:442. doi: 10.1186/s12859-020-03773-2 (PMC7542696; doi:10.1186/s12859-020-03773-2)
Supplement: Supplementary file 1 — Additional file 1: This PDF file contains the supplementary figures referred throughout the manuscript. [file 12859_2020_3773_MOESM1_ESM.pdf]

Supplementary Figures

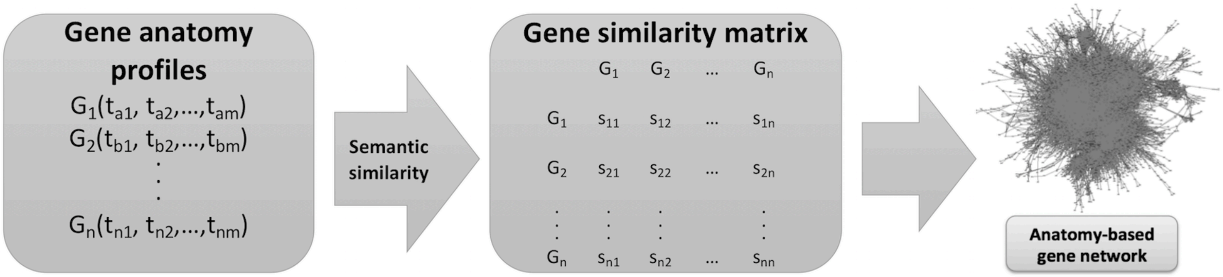

Fig. S1. The general workflow for generating anatomy-based gene networks. The genes are represented by G_1, G_2, etc., and their anatomical entities (Uberon terms) are represented by t_{a1}, t_{b1}, etc. In the gene similarity matrix, the similarity scores between genes are represented by s_{11}, s_{12}, etc.

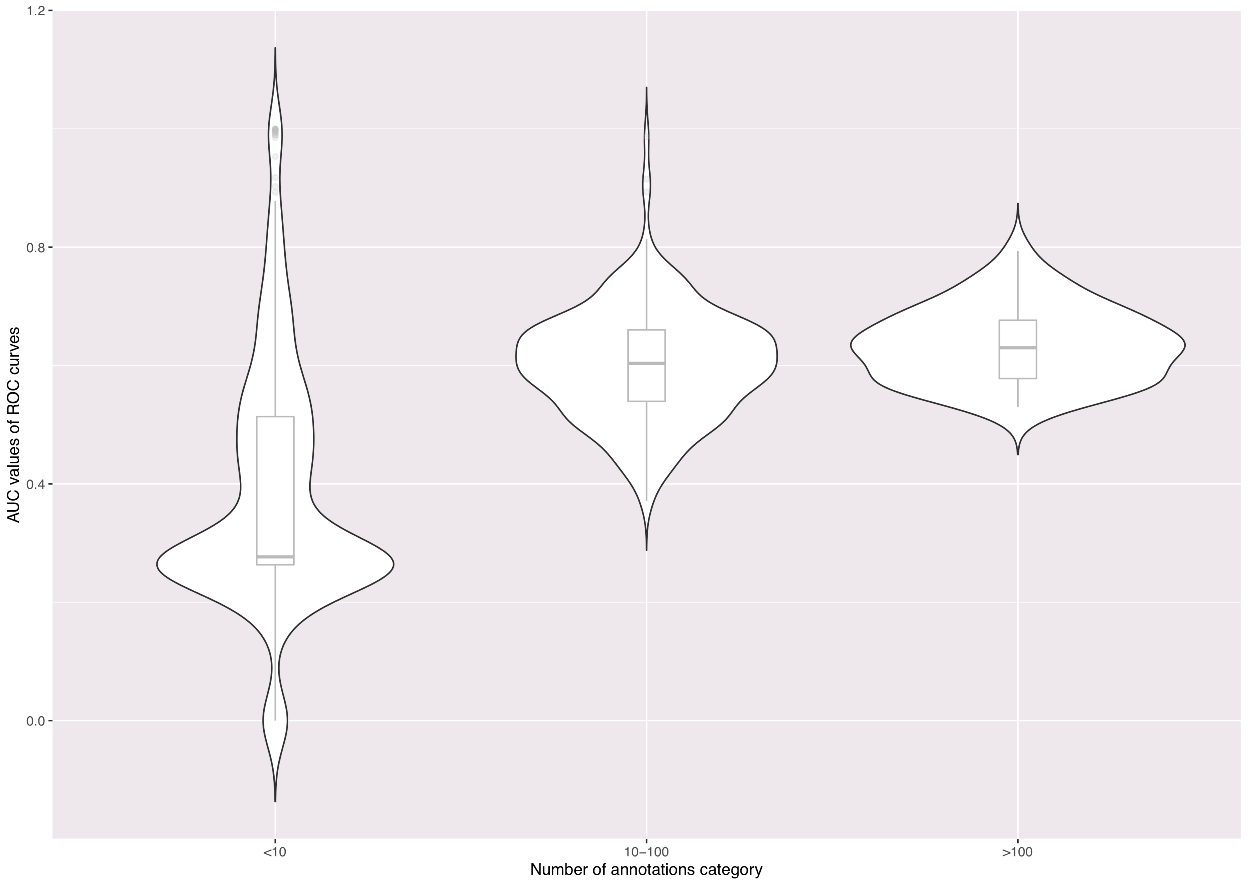

Fig. S2. The effect of the number of gene annotations of anatomical entities on ROC performance. The violin plot comparison of AUC distributions of ROC curves for anatomical entities with less than 10, 10-100, above 100 gene annotation categories. The ROC curves were generated by evaluating the zebrafish PPI network.

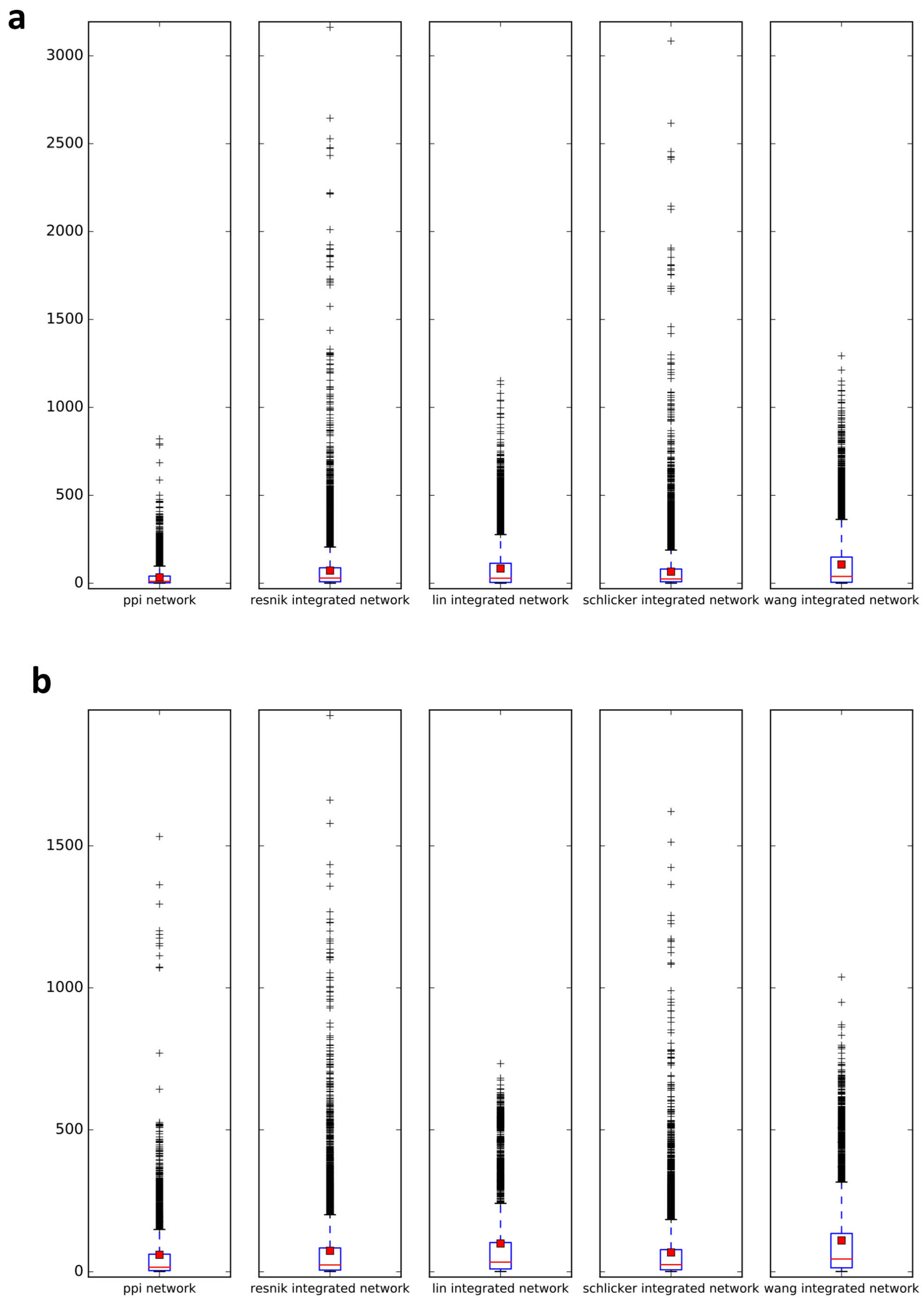

Fig. S3. Comparisons of degree distributions between PPI networks and integrated networks. The integrated networks constructed using different semantic similarity calculation methods are compared with PPI networks for (a) zebrafish and (b) mouse.

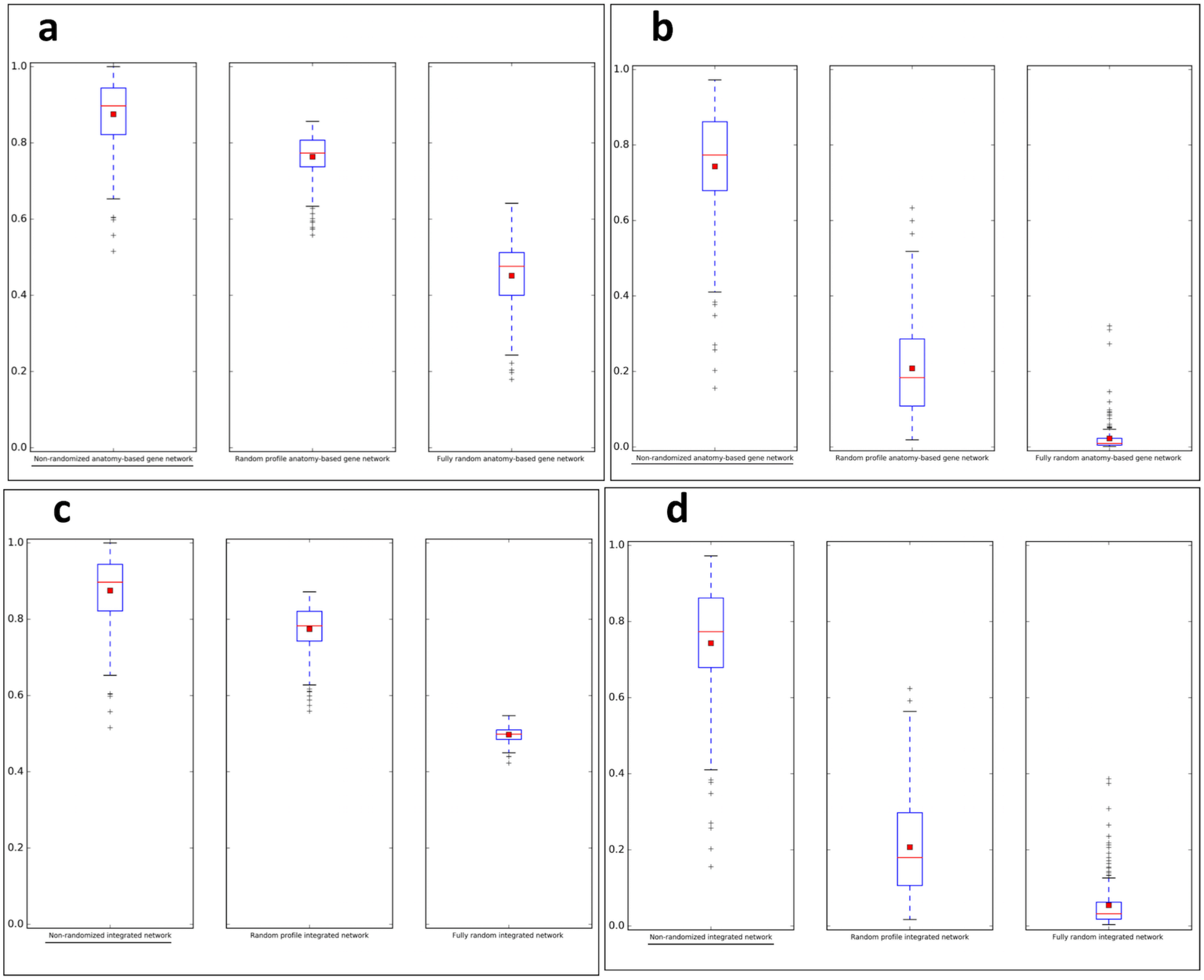

Fig. S4. The network performance comparisons between non-randomized and randomized networks for mouse. The boxplot comparisons of the AUC distributions for (a) ROC and (b) precision-recall curves for the filtered non-randomized anatomy-based gene network, randomized profile anatomy-based gene network, and fully randomized anatomy-based gene network for the Wang method for mouse. The boxplot comparisons of the AUC distributions for (c) ROC and (d) precision-recall curves for the filtered non-randomized integrated network, randomized profile integrated network, and fully randomized integrated network for the Wang method for mouse. In the boxplots, the red line and the square represent the median and mean, respectively, and the name of the best performing network is underlined.

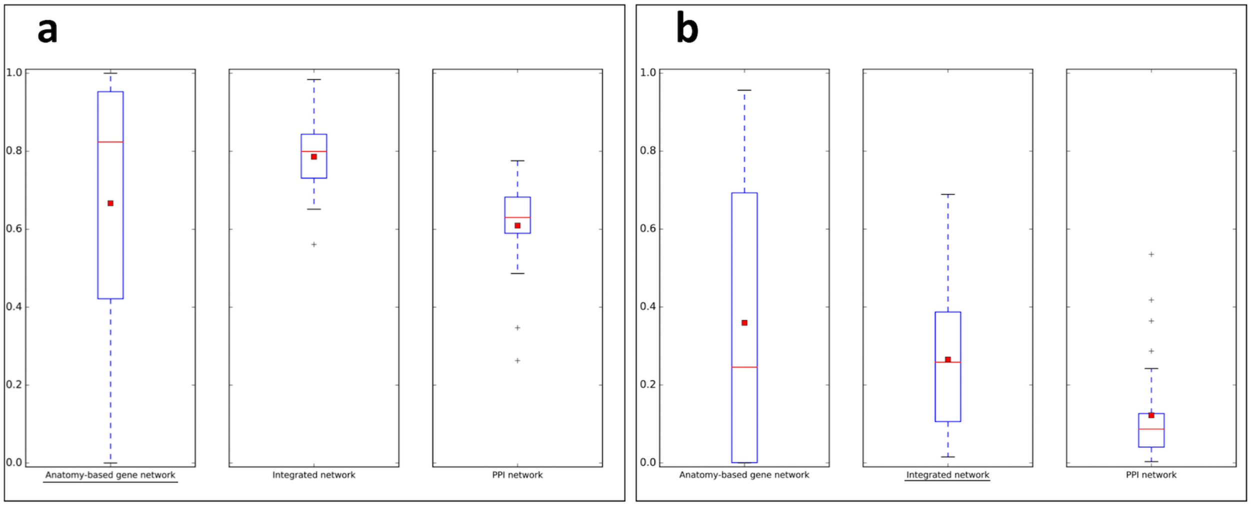

Fig. S5. The network performance comparisons for mouse networks when evaluated by randomly removed 30 anatomical entities. The boxplot comparisons of the AUC distributions for (a) ROC and (b) precision-recall curves for the filtered integrated network, PPI network, and anatomy-based gene network for the Wang method for mouse. The integrated network and the anatomy-based gene network were generated using the mouse anatomy profiles after randomly removing 30 anatomical entities, which had at least 10 gene annotations. The same 30 entities were used for the evaluation. In the boxplots, the red line and the square represent the median and mean, respectively, and the name of the best performing network is underlined.

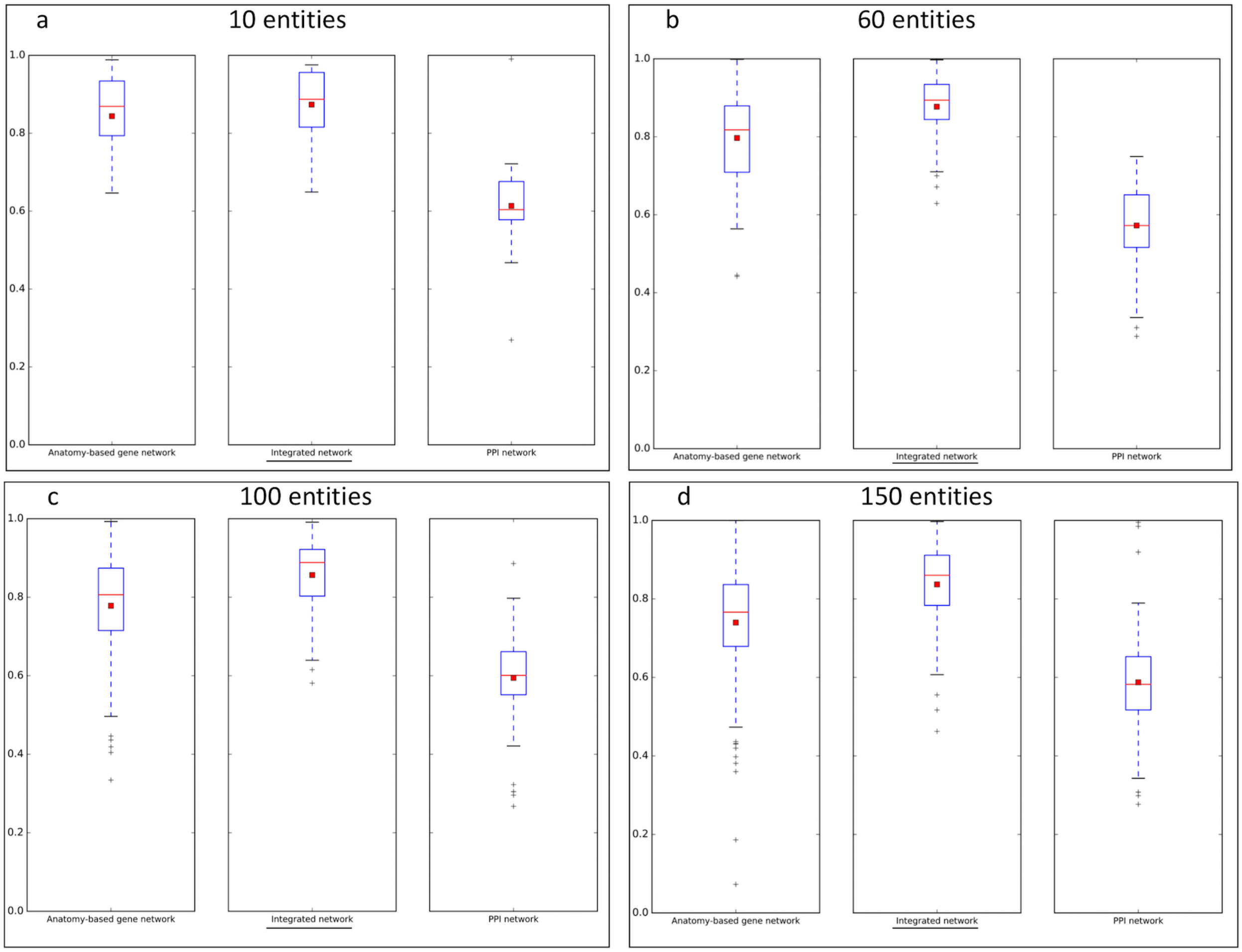

Fig. S6. The ROC performance comparisons for zebrafish when evaluated by different numbers of removed anatomical entities. Each boxplot comparison compares the AUC distributions of ROC curves for the filtered integrated networks, PPI networks, and anatomy-based gene networks for the Wang method for zebrafish. The integrated networks and the anatomy-based gene networks were generated using the zebrafish anatomical profiles after randomly removing (a) 10, (b) 60, (c) 100, and (d) 150 anatomical entities, which had at least 10 gene annotations. The same removed entities were used for the evaluation for each comparison. In the boxplots, the red line and the square represent the median and mean, respectively, and the name of the best performing network is underlined.

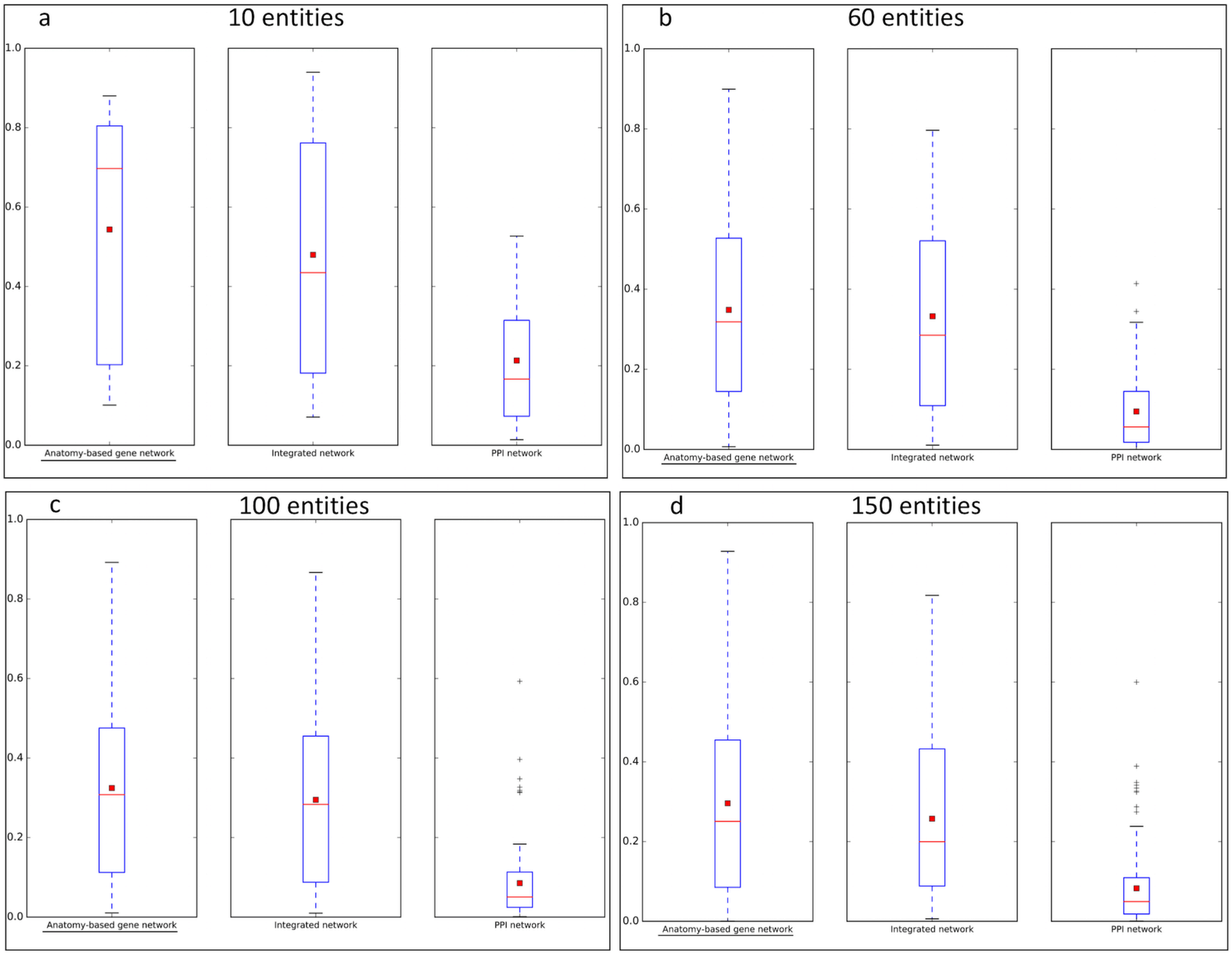

Fig. S7. The precision-recall performance comparisons for zebrafish when evaluated by different numbers of removed anatomical entities. Each boxplot comparison compares the AUC distributions of precision-recall curves for the filtered integrated networks, PPI networks, and anatomy-based gene networks for the Wang method for zebrafish. The integrated network and the anatomy-based gene network were generated using the zebrafish anatomy profiles after randomly removing (a) 10, (b) 60, (c) 100, and (d) 150 anatomical entities, which had at least 10 gene annotations. The same removed entities were used for the evaluation for each comparison. In the boxplots, the red line and the square represent the median and mean, respectively, and the name of the best performing network is underlined.

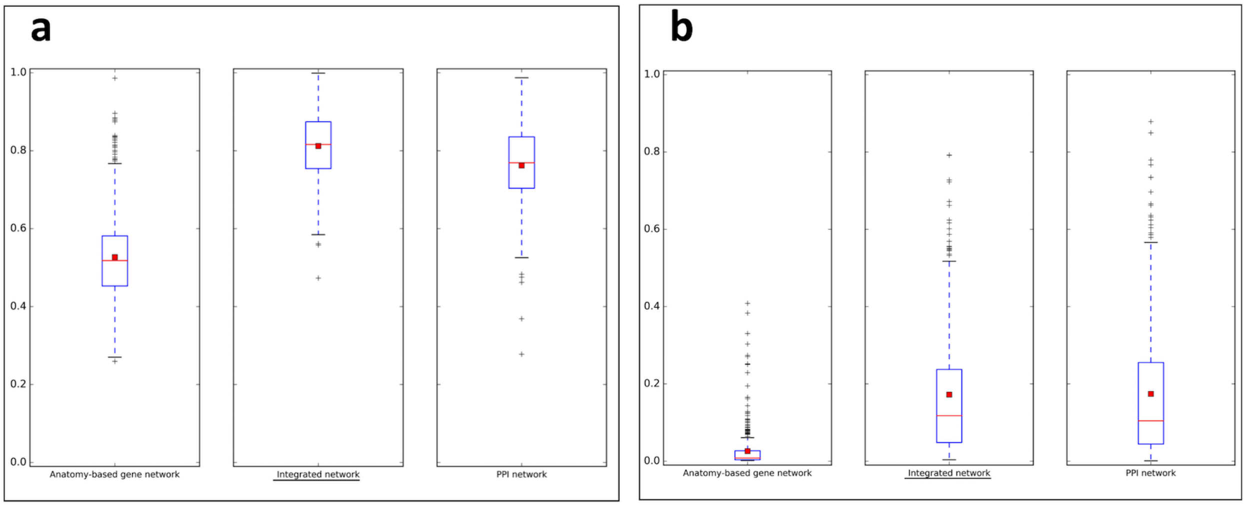

Fig. S8. The network performance comparisons for mouse networks when evaluated by Gene Ontology-Biological Process (GO-BP) entities. The boxplot comparisons of the AUC distributions of (a) ROC and (b) precision-recall curves for the filtered integrated network, PPI network, and anatomy-based gene network for the Wang method in mouse. The networks were evaluated using the annotation profiles containing GO-BP entities for the mouse genes. In the boxplots, the red line and the square represent the median and mean, respectively, and the name of the best performing network is underlined.
