# Supplementary material for: Integration of anatomy ontology data with protein–protein interaction networks improves the candidate gene prediction accuracy for anatomical entities
Source: BMC Bioinformatics. 2020 Oct 7;21:442. doi: 10.1186/s12859-020-03773-2 (PMC7542696; doi:10.1186/s12859-020-03773-2)
Supplement: Supplementary file 3 — Additional file 3: This PDF file contains the definitions of the four semantic similarity calculation methods (Wang method, Resnik method, Lin method, and Schlicker method) used for anatomy-based gene network and integrated network construction. [file 12859_2020_3773_MOESM3_ESM.pdf]

The definitions for the semantic similarity calculation methods

The semantic similarity equations for Resnik, Lin, and Schlicker methods are given below (equations 1, 2, and 3).

Resnik:

$$sim_R\,(t_1, t_2) = \max_{t \in S(t_1,t_2)} \{IC(t)\} \quad (1)$$

Lin:

$$sim_L\,(t_1, t_2) = \max_{t \in S(t_1,t_2)} \left\{\frac{2IC(t)}{IC(t_1)+IC(t_2)}\right\} \quad (2)$$

Schlicker:

$$sim_S\,(t_1, t_2) \;=\; \max_{t \in S(t_1,t_2)} \left\{\frac{2IC(t)}{IC(t_1)+IC(t_2)}\;(1 + IC(t))\right\} \quad (3)$$

In the above equations, t_1 and t_2 represent the ontology terms between which the similarity is calculated, whereas *S* denotes the set of common ancestors for the two terms. The information content for a given term *t* is represented by *IC(t)*, which is calculated based on the number of genes annotated to the term *t* as illustrated below (equations 4 and 5).

$$IC(t) = \,-log\;(p(t)) \quad (4)$$

$$P(t) = \frac{Number\ of\ genes\ associated\ with\ the\ term\ t}{Total\ number\ of\ genes\ associated\ with\ the\ entire\ ontology} \quad (5)$$

The Wang method does not use the IC. It only depends on the ontology structure and the relationships between the entities. The equation for the Wang semantic similarity calculation between the two entities t_1 and t_2 is given below.

Wang:

$$sim_W(t_1, t_2) = \frac{\sum_{t \in T_1 \cap T_2}(S_{t_1}(t) + S_{t_2}(t))}{SV(t_1) + SV(t_2)} \quad (6)$$

In the above equation, $S_{t_i}(t)$ represents the semantic contribution of term *'t'* on term t_i, when *'t'* is an ancestor of t_i. The term $SV(t_i)$ represents the semantic contribution of all the ancestors of term t_i on itself.
